# Identification of QTLs and critical genes related to sugarcane mosaic disease resistance

**DOI:** 10.3389/fpls.2023.1107314

**Published:** 2023-02-02

**Authors:** Guilong Lu, Zhoutao Wang, Yong-Bao Pan, Qibin Wu, Wei Cheng, Fu Xu, Shunbin Dai, Boyu Li, Youxiong Que, Liping Xu

**Affiliations:** ^1^ Key Laboratory of Sugarcane Biology and Genetic Breeding, Ministry of Agriculture and Rural Affairs, Fujian Agriculture and Forestry University, Fuzhou, China; ^2^ Institute of Vegetables, Tibet Academy of Agricultural and Animal Husbandry Sciences, Lhasa, China; ^3^ USDA-ARS, Sugarcane Research Unit, Houma, LA, United States

**Keywords:** sugarcane (*Saccharum* spp. hybrids), sugarcane mosaic disease, QTL mapping, gene mining, expression profiles

## Abstract

Mosaic viral diseases affect sugarcane productivity worldwide. Mining disease resistance-associated molecular markers or genes is a key component of disease resistance breeding programs. In the present study, 285 F_1_ progeny were produced from a cross between Yuetang 93-159, a moderately resistant variety, and ROC22, a highly susceptible variety. The mosaic disease symptoms of these progenies, with ROC22 as the control, were surveyed by natural infection under 11 different environmental conditions in the field and by artificial infections with a mixed *sugarcane mosaic virus* (SCMV) and *sorghum mosaic virus* (SrMV) inoculum. Analysis of consolidated survey data enabled the identification of 29 immune, 55 highly resistant, 70 moderately resistant, 62 susceptible, and 40 highly susceptible progenies. The disease response data and a high-quality SNP genetic map were used in quantitative trait locus (QTL) mapping. The results showed that the correlation coefficients (0.26~0.91) between mosaic disease resistance and test environments were significant (*p*< 0.001), and that mosaic disease resistance was a highly heritable quantitative trait (*H^2^* = 0.85). Seven mosaic resistance QTLs were located to the SNP genetic map, each QTL accounted for 3.57% ~ 17.10% of the phenotypic variation explained (PVE). Furthermore, 110 pathogen response genes and 69 transcription factors were identified in the QTLs interval. The expression levels of nine genes (*Soffic.07G0015370-1P*, *Soffic.09G0015410-2T*, *Soffic.09G0016460-1T*, *Soffic.09G0016460-1P*, *Soffic.09G0017080-3C*, *Soffic.09G0018730-3P*, *Soffic.09G0018730-3C*, *Soffic.09G0019920-3C* and *Soffic.03G0019710-2C*) were significantly different between resistant and susceptible progenies, indicating their key roles in sugarcane resistance to SCMV and SrMV infection. The seven QTLs and nine genes can provide a certain scientific reference to help sugarcane breeders develop varieties resistant to mosaic diseases.

## Introduction

Sugarcane mosaic disease (SMD) is a worldwide issue that has long plagued sugarcane production. The disease is mainly caused by single or co-infection of *Sugarcane mosaic virus* (SCMV), *Sorghum mosaic virus* (SrMV), and *Sugarcane streak mosaic virus* (SCSMV) (Lu et al., 2021). SMD exhibiting typical “mosaic” symptoms (Grisham, 2011) can seriously reduce the photosynthetic capacity (Bagyalakshmi et al., 2019), yield, and quality of sugarcane (Singh et al., 2003; Viswanathan and Balamuralikrishnan, 2005). Pandemic SMD has occurred many times in history and caused huge economic losses and even bankruptcies to many sugar companies (Koike and Gillaspie, 1989; Grisham, 2011). Breeding and rationally planting of SMD-resistant varieties are the most economical and effective methods to prevent and control the disease.

So far, both natural infection disease surveys and artificial inoculation-induced infection disease surveys are used in SMD resistance assessments. Using the natural infection method, Li et al. (2013); Da-Silva et al. (2015a); Yang et al. (2020), and Lavín-Castaeda et al. (2020) successively screened sugarcane breeding materials, cultivars, or hybrid offspring populations. A few varieties (lines) with immunity or good resistance to SMD provided good material for mosaic disease resistance gene mining and hybrid breeding. Although this method is simple and saves labor and time, it requires a high level of professional ability and is often affected by environments. Alternatively, several artificial inoculation methods, including friction (Da-Silva et al., 2015b; De-Souza et al., 2017), spray (Dean, 1960), stalk cutting (Li et al., 2013; Li et al., 2018), and injection inoculations (Zhou, 2015), can be well controlled and be evaluated under a set stress. Roossinck (2015) assumed that the occurrence and prevalence of plant diseases depended on a compound effect among host plants, pathogens, and environmental factors. Therefore, it is of vital importance to choose the most suitable growth stage and the optimum inoculation methods for improved accuracy of resistant phenotype identification during field evaluation.

The development of practical molecular markers and related detection methodology are the basis for molecular marker-assisted breeding. Currently, traditional DNA markers, such as amplified fragment length polymorphism (AFLP), restriction fragment length polymorphism (RFLP), and simple sequence repeats (SSR), are being used in quantitative trait locus (QTL) mapping or bulk segregant analysis (BSA) research (Xia et al., 1999; Duble et al., 2000; Xu et al., 2000; Dussle et al., 2003; Yuan et al., 2004). Several SCMV-resistance markers were identified in corn (*Zea mays* L., 2*n* = 2*x* = 20; genome size ~2,300 Mb) (Schnable et al., 2009). Single nucleotide polymorphisms (SNP) markers are superior markers due to wide distribution, huge quantity, high stability, strong representativeness, and bi-allelicity (Rafalski, 2002). SNP chips represent a high-throughput, automated, and relatively cost-effective genotyping method (Laframboise, 2009), which has been used to identify resistance genes to *Bean common mosaic virus* in soybean (2*n* = *2x* = 40) (Bello et al., 2014) and to *Soil-borne wheat mosaic virus* in wheat (*2n* = 6*x* = 42) (Liu et al., 2014). However, due to the complexity of the sugarcane genome (2*n* = 12*x* = 100~130 and genome size ~10 Gb) (Roach, 1989; D'Hont et al., 1998), sequencing technology, and high cost, only two SNP chips, namely, the 345K chip of Aitken et al. (2017) and the 100K chip of You et al. (2019), have been developed in sugarcane. The 100K SNP chip has a polymorphism rate of up to 77.04% and has been successfully used in QTL mapping of disease resistance markers to yellow leaf disease (You et al., 2019), ratoon stunting disease (You et al., 2020), and leaf blight disease (Wang et al., 2021) in sugarcane.

In plants, compared to qualitative resistance traits, quantitative resistance traits are more broad-spectrum and persistent and play an important role in preventing large-scale disease outbreaks due to the loss of a single gene resistance (Poland et al., 2009). For instance, a QTL locus *qMdr9.02* was found to be associated with resistance to southern leaf blight, northern leaf blight, and gray leaf spot in maize (Yang et al., 2017). However, to date, only four SCMV resistance-associated markers (*AFLP-346*, *AFLP-372*, *AFLP-538*, and *CV29.13*), each accounting for 5.51 to 14.02% of PVE, were reported by Burbano et al. (2022). The objectives of this study were to construct a genetic mapping population, to evaluate the SMD response of the mapping population, and to develop SMD resistance-associated QTL markers and suggest candidate genes for the improvement of the efficiency and accuracy of sugarcane breeding.

## Materials and methods

### Plant material and field planting

**Figure 1:**
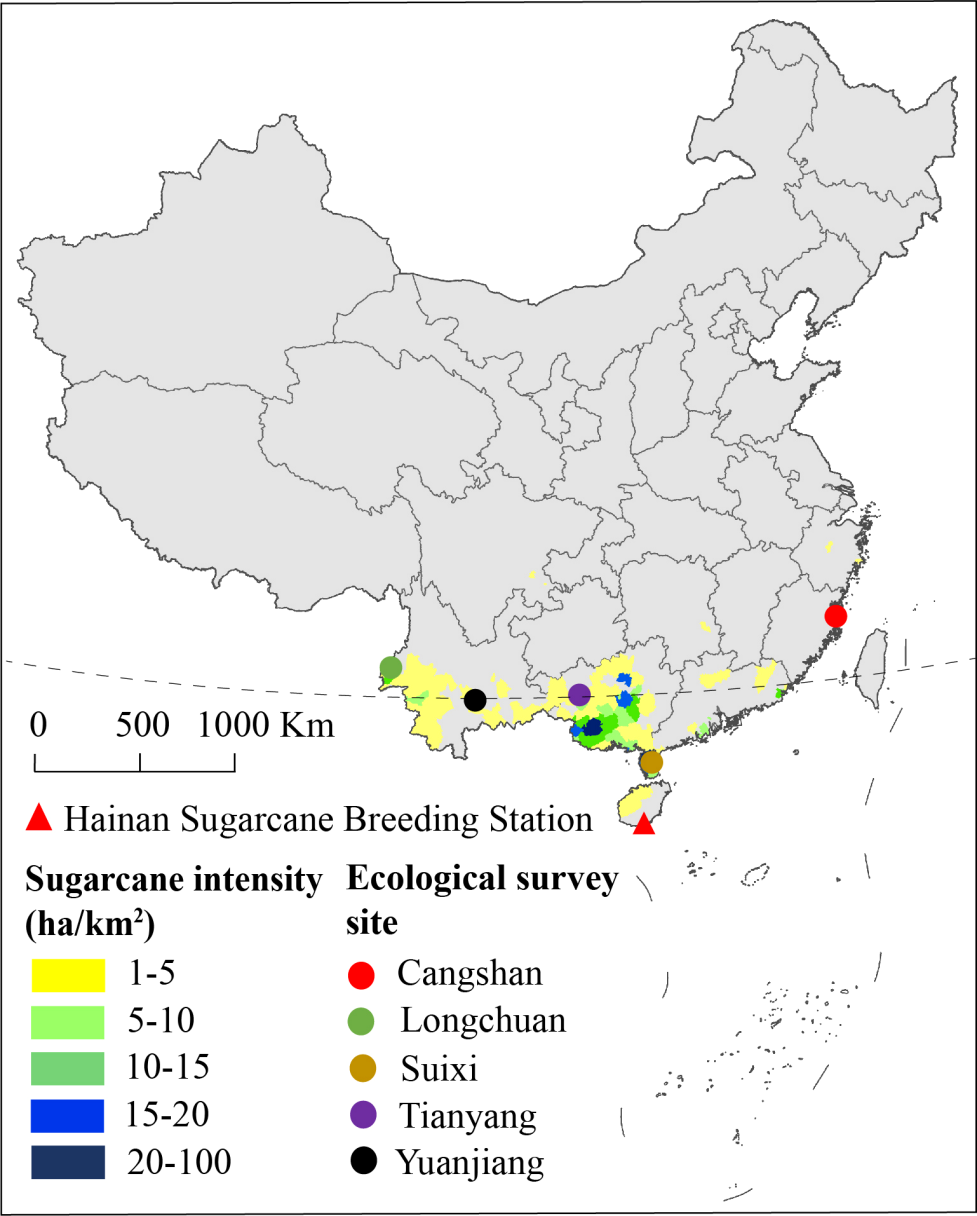
Ecological survey sites and sugarcane crop density in China based on the data from the 2020 Statistical Yearbook.

Two hundred and eighty-five F_1_ progeny were produced from a cross between YT93-159 (moderately resistant to SMD) and ROC22 (highly susceptible to SMD). The cross was made in 2014 at the Hainan Sugarcane Breeding Station, Yacheng, Hainan, China. After vegetative propagation, stems of these progeny were planted at five different ecological sites, namely, Cangshan (119˚14’E, 26˚5’N), Longchuan (97˚53’E, 24˚15’N), Suixi (110˚10’E, 21˚6’N), Tianyang (107˚0’E, 23˚39’N), and Yuanjiang (101˚59’E, 23˚36’N) (**Figure 1**; **Supplementary Table 1**). A randomized block design was adopted for field planting. Specifically, the trial design in Cangshan and Longchuan contained three replications, Suixi and Yuanjiang contained two replications, and Tianyang contained one replicate. Specific row spacing and planting density were shown in **Supplementary Table 2**. The five ecological sites were routinely managed according to conventional planting measures, and stalk-cutting was done at the end of December each year.

### Mosaic disease survey

#### By natural infection

To identify the appropriate survey season, SMD symptoms on a field grown, highly susceptible progeny FN14-255 were monitored monthly on the campus of Fujian Agriculture and Forestry University (FAFU) (119˚14’E, 26˚5’N). Three typical +1 leaves were sampled for comparison. The three periods showing the most severe symptoms were selected for investigating natural SMD incidence.

#### By artificial inoculation

Before planting, a machete was used to cut the stem of FN14-255 into single-bud pieces, which were rinsed in running water overnight. Only single-bud pieces that met the criteria of 1) having one full and healthy bud, and 2) with flat incisions without any cracks were kept. A super constant temperature tank (Ningbo Prandt Instrument Co., Ltd, Ningbo, China) was used for hot water treatment. Water temperature was set and kept at ± 0.2°C of 50°C (CK), 55°C, 57°C, 59°C, and 61°C. Water level was maintained at about 2/3 tank full. Treatment was for 30 minutes. Once the treatment was completed, the stems were rinsed in running water until the buds cooled completely. The buds were cultured in a greenhouse (**Supplementary Figure 1**) under 12 h light/12 h dark with a light intensity of 15,000 Lx and a relative humidity of 60%. Greenhouse temperature was set to 28°C before inoculation and 25°C after inoculation. Each treatment had 30 buds with three replications. After 30 d, the one-step multiplex reverse transcription PCR (RT-PCR) method of Shan et al. (2020) was used to detect different sugarcane mosaic virus. The oligonucleotide sequence of species-specific RT-PCR primers and the length of targeted fragments are shown in **Table 1**.

**Table 1:** Species-specific RT-PCR primers for the detection of three sugarcane mosaic viruses.

Virus	Primer sequence (5’→3’)	Annealing temperature (°C)	Amplification size (bp)
SCMV	F: GCGCGGTATGCATTTGACTT	58	200
	R: CACTCCCAACAGAGAGTGCAT		
SrMV	F: AACAGGATGCCGATGCGAAA		450
	R: CGTTGATGTTCGGTGAGCAA		
SCSMV	F: GAACGCAGCCACCTCAGAAT		800
	R: CCAAAATGAGCGCCTCCGAT		

The method of Li et al. (2013) was used to configure the viral inoculum mixture. The viral source was SMD symptomatic leaves from sugarcane variety Funong 41 that was planted on the Sugarcane Farm on the campus of FAFU. SCMV and SrMV pathogens were detected in these leaves by RT-PCR (**Supplementary Figure 2**). YT93-159 and ROC22 were used to test different inoculation methods, including spray, micro-injection, quartz sand friction, abrasive cloth friction, rasp friction, young stem cut, single bud soaking, single bud soaking and quartz sand friction (**Supplementary Figure 3**, **Supplementary Table 3**), and to choose the best inoculation method to inoculate the test population. In 2021, three batches of viral inoculums were administered successively. One was conducted at the sugarcane station of FAFU during February to April. Another was conducted in a climate-controlled greenhouse of the Key Laboratory of Sugarcane Biology and Genetic Breeding, Ministry of Agriculture and Rural Affairs, FAFU from May to July. A final inoculation was conducted in the same greenhouse from October to December. For each genotype, 15 single buds were inoculated with three replications and were kept in the dark for 24 h after inoculation. Four weeks post inoculation, SMD incidence was investigated for three consecutive sessions with an interval of one week.

### Resistance evaluation

One growth cycle at one ecological site and a batch of artificial inoculation treatments were considered as one environment. The highest SMD incidence rate out of the three surveys was used to determine the level of SMD resistance for each F_1_ progeny in a single environment. Comprehensive evaluation was based on the maximum value of resistance across multiple natural and artificial inoculation infection environments. The SMD grading system was set according to the method of Li et al. (2000) (**Table 2**). During comprehensive evaluation, if the disease incidence rate of ROC22 (control) in an environment was more than 66.01%, the external SMD stress was considered sufficient, and the survey data valid. If the disease incidence rate of ROC22 (control) in an environment was less than 66.01%, then the external SMD stress was assumed to be insufficient, and the environmental data discarded. The following formula was used to calculate SMD incidence rate (%):

**Table 2:** Resistance grading based on SMD incidence.

Grade	Resistance	SMD Incidence (%)
1	Immune	0
2	Highly resistant	0.01~10.00
3	Moderately resistant	10.01~33.00
4	Susceptible	33.01~66.00
5	Highly susceptible	66.01~100

SMD incidence rate (%) = number of diseased plants/total number of plants per F_1_ progeny × 100%.

### Correlation analysis and generalized heritability estimation

The QTL IciMapping V4.2 software (Chinese Academy of Agricultural Sciences, Beijing, China) was used to analyze the correlation and calculate the generalized heritability (*H^2^*) using the following calculation formula:


, $$H^{2}=\sigma_{g}^{2}/(\sigma_{g}^{2}+\frac{\sigma_{ge}^{2}}{n}+\frac{\sigma_{e}^{2}}{nr}),$$

Where $\sigma_{g}^{2}$ is genotype variance, $\sigma_{e}^{2}$ is error variance, $\sigma_{ge}^{2}$ is genotype-by-environment interaction variance, *n* is the number of environments; and *r* is number of survey periods within each environment.

### QTL mapping

The SMD resistance grading data of the F_1_ progeny population and the sugarcane 100K SNP chip-based genetic map (**Supplementary Table 4**) (Wang et al., 2021) were used to conduct QTL mapping using the inclusive composite interval mapping (ICIM) of GACD 1.2 software (Chinese Academy of Agricultural Sciences, Beijing, China), with a logarithm of odds (LOD) threshold of 2.5 and other default parameters. Loci with ≥ 10% phenotypic variation explained (PVE) values were defined as major QTLs, and loci with< 10% PVE were minor QTLs. QTLs were named according to McCouch et al. (1997) with “*q*” plus the sugarcane mosaic disease resistance (Rsm) trait, followed by linkage group number in italics. R software (R-Tools Technology Inc., Ontario, Canada), Origin 9.0 software (OriginLab Inc., Massachusetts, USA), and Adobe Illustrator CS6 software (Adobe Systems Inc., California, USA) were used to draw the position of QTL on the linkage group.

### Candidate gene mining

The protein sequences of all genes in the QTL interval were extracted according to the GFF annotation file of a *Saccharum officinarum* genome (http://sugarcane.zhangjisenlab.cn/sgd/html/-index.html). The Plant Pathogen Receptor Genes database (PRGdb 4.0, http://prgdb.org/prgdb4/) was used to search for genes related to disease resistance. At the same time, disease resistance-related transcription factors were extracted from the plant transcription factor database (TFDB 5.0, http://planttfdb.gao-lab.org/index.php) (Osuna-Cruz et al., 2018).

### Critical gene and functional structure prediction

Stems of Yuetang 93-159, ROC22, five immune, and five highly susceptible progeny were detoxified in a hot water bath as previously described. Plants with 2~3 fully expanded leaves from the detoxified buds were inoculated with a mixed inoculum of SCMV and SrMV by quartz sand friction. Leaf samples were taken on 0 d, 1 d, and 4 d post inoculation, RT-PCR was conducted to detect the viruses at 4 d post inoculation (**Supplementary Figure 4**). There were four plants in each of the three biological replicates. RNA was extracted by the Trizol method, and the integrity of the extracted RNA samples was checked using an Agilent 2100 Bioanalyzer (Agilent Technologies, Santa Clara, CA, USA). The integrity number of a qualified RNA sample was considered greater than 6.0, and the detection quality was A-level (**Supplementary Table 5**). The qualified RNA samples were sent to Novogene Bioinformatics Technology Co., Ltd. (Beijing, China) for transcriptome sequencing. The DNBSEQ-T7 (Shenzhen Huada Intelligent Technology Co., Ltd., Shenzhen, China) sequencing platform was used for paired-end sequencing, and each library yielded ≥ 12 Gb of sequence data (**Supplementary Table 6**). The Transcripts Per Kilobase Million (TPM) normalization method (Wang et al., 2021) was used to calculate the expression levels of all genes. TBtools V1.0986 software (South China Agricultural University, Guangzhou, China) was used to draw an expression heat map of candidate genes, and to locate significantly differentiated key genes in the *S. officinarum* genome. An online tool GSDS V2.0 (http://gsds.gao-lab.org/) was used to describe the gene structure. The *Arabidopsis* genome (https://www.arabidopsis.org/Blast/index.jsp) was referred for functional annotation with e-value threshold set to 1e^-10^.

### Data statistics and analysis

A Canon EOS 60D camera (Canon Inc., Tokyo, Japan) was used to capture the images of SMD symptoms. Data were achieved as Excel 2010 (Microsoft Inc., Washington, USA) spreadsheets. Duncan’s significant difference test and descriptive statistics were performed using IBM SPSS^®^ V25 software (International Business Machines Inc., California, USA).

## Results

### Phenotypic analysis and evaluation

#### Determination of the natural survey period

**Figure 2:**
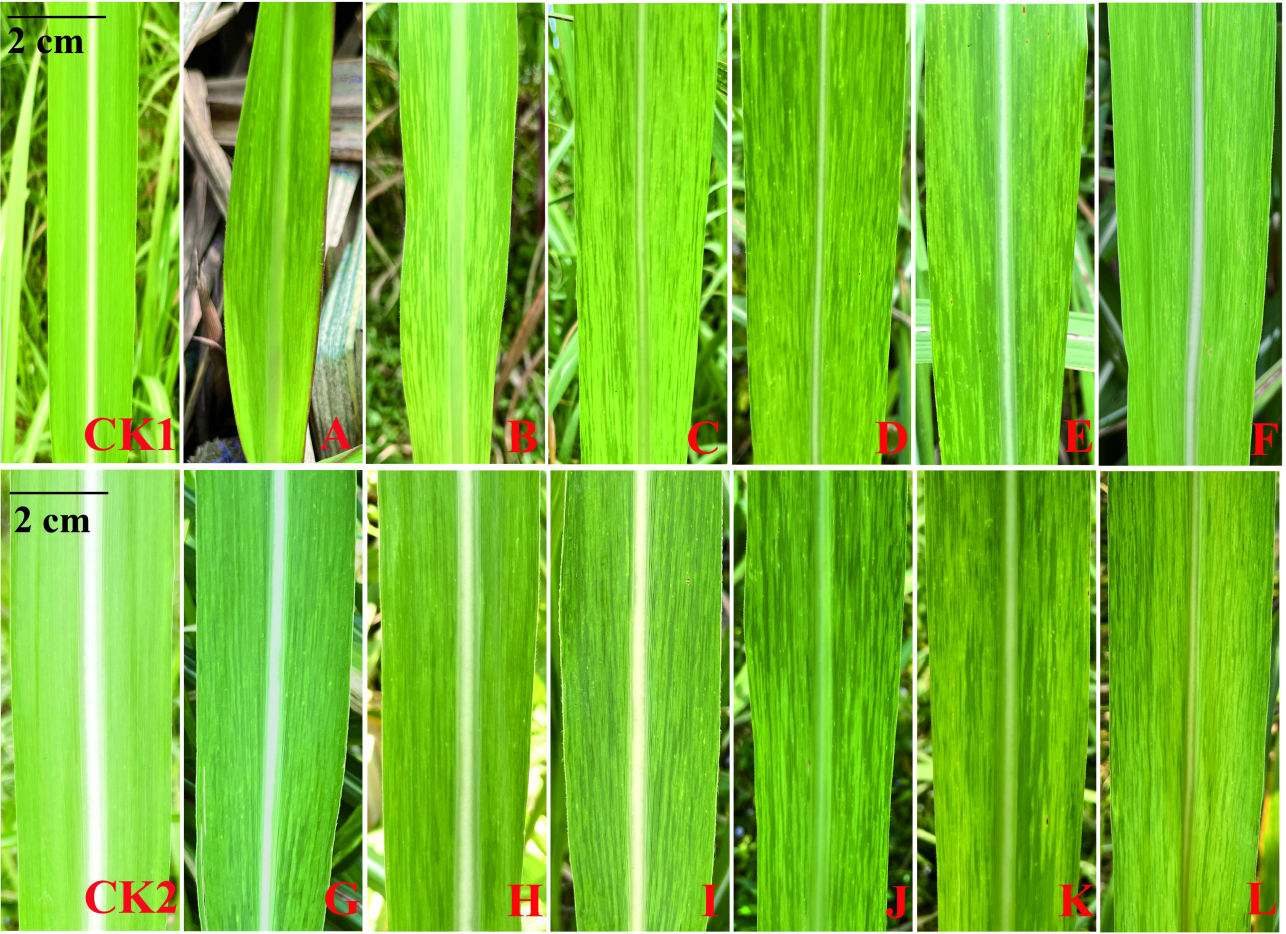
SMD symptoms of a highly susceptible progeny (FN14-255) observed in different months CK1: disease free control (January); CK2: disease free control (December); **(A–L)**: January-December, respectively.

The SMD symptoms of a highly susceptible progeny (FN14-255) are shown in **Figure 2**. The figure shows the symptoms of infected sugarcane leaves were more clearly distinguishable during February to April and October to December, with mosaic symptoms covering the entire leaf. Nevertheless, the symptoms were significantly weakened in January and in May to September, especially from June to August, the symptoms were suppressed by high temperature, and can only be observed at the bottom of the leaves. Therefore, the field natural incidence survey was arranged in March, April and November, respectively.

#### Hot-water detoxification and artificial inoculation

Germination time was obviously delayed, and germination rate was significantly reduced with increasing hot water temperature (**Supplementary Table 7**). On the other hand, mild leaf symptoms could be seen from the 50°C treatment. And even barely visible from the 55°C treatment. However, no symptom was observable from the 57°C, 59°C, and 61°C treatments. As shown in **Supplementary Figure 5**, no band was visible on the gels, indicating that all three target viruses were not detectable for the samples treated at 59°C and 61°C. Therefore, a hot water treatment at 59°C for 30 min can completely detoxify the viruses, albeit with a germination rate of about 30% (**Supplementary Table 7**). The ‘single bud soaking + quartz sand friction’ method had the highest inoculation efficiency (**Supplementary Table 8**). Therefore, this method was used to inoculate the mapping population material.

#### Comprehensive evaluation

**Figure 3:**
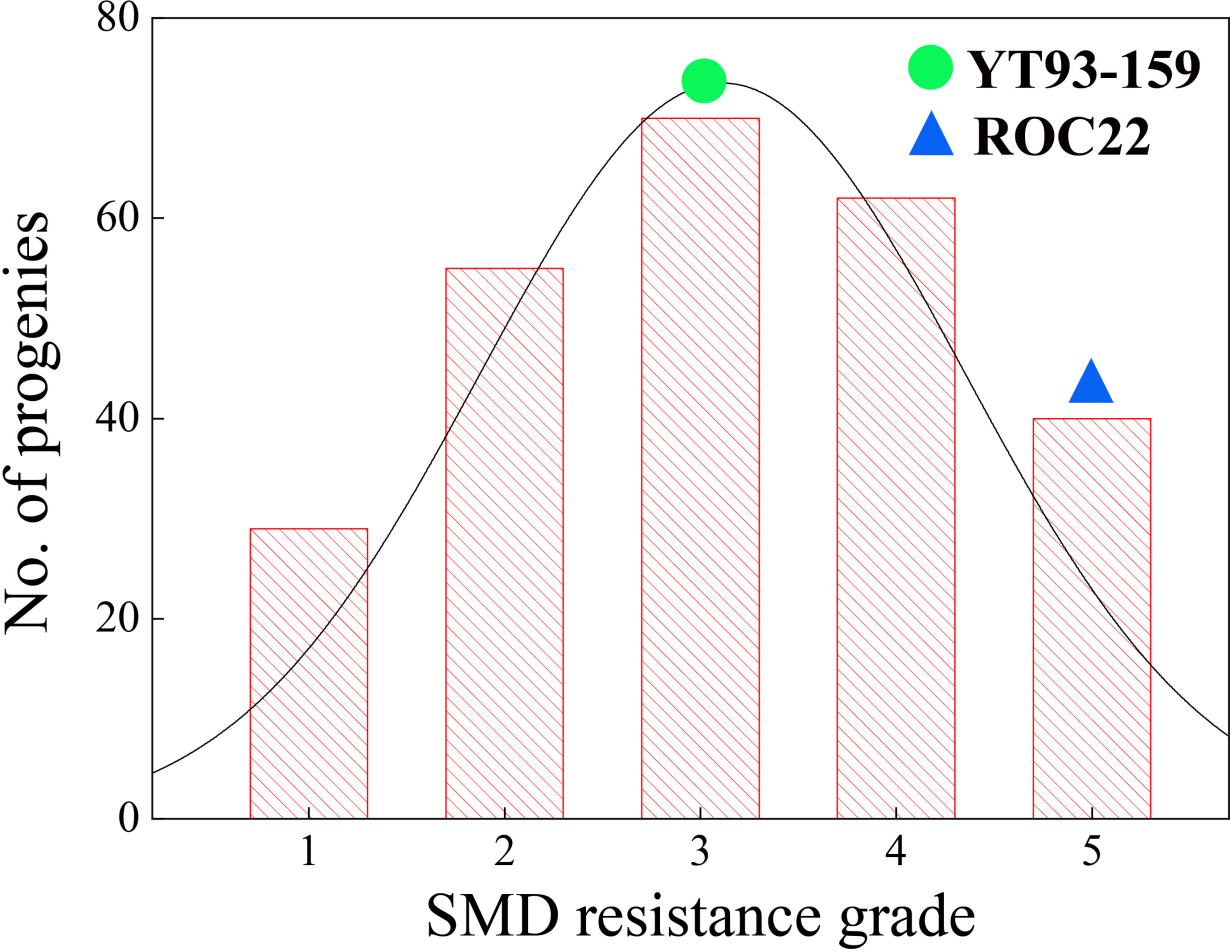
Distribution of five SMD resistance grades (**Table 2**) within a sugarcane mapping population YT93-159 belongs to Grade 3 and ROC22 belongs to Grade 5.

The SMD survey data for the F_1_ mapping population from 11 natural infection and 3 artificial inoculation infection environments during 2020 to 2022 are shown in **Supplementary Table 9**. The frequency distribution of SMD resistance grades within this population in 14 environments is shown in **Supplementary Figure 6**. The data from two environments at Cangshan ecological site (block 2) were excluded from the comprehensive evaluation and resistance analysis due to insufficient pathogen stress. Accordingly, the population was comprehensively evaluated based on nine natural environments and three artificial inoculation environments. Among the 285 progenies, 29 immune, 55 highly resistant, 70 resistant, 62 susceptible, and 40 highly susceptible progenies were identified. The remaining 29 progenies had inconsistent SMD responses. The SMD resistance trait segregated widely within the F_1_ mapping population and showed an obvious hybrid vigor (Heterosis) phenomenon (**Figure 3**). That was in line with the typical characteristics of a quantitative trait, indicating its suitability for QTL analysis.

### Correlation analysis and generalized heritability

Certain differences of SMD incidence were observed in the mapping population across different environments. For example, SMD incidence in the ratoon crop was significantly higher than the plant cane crop. The SMD tended to accumulate when the sugarcane crop underwent prolonged ratooning. Correlation coefficients between the resistance trait and different environments were 0.26~0.91 (**Supplementary Table 10**), all these values were very significant (*p*< 0.001), indicating that the SMD resistance was a stable trait. Not surprisingly, the estimated broad sense heritability (*H^2^*) of SMD resistance in this mapping population under 14 environments was 0.85, which implied that the SMD resistance trait was mainly determined by genetic factors.

### QTL mapping

**Figure 4:**
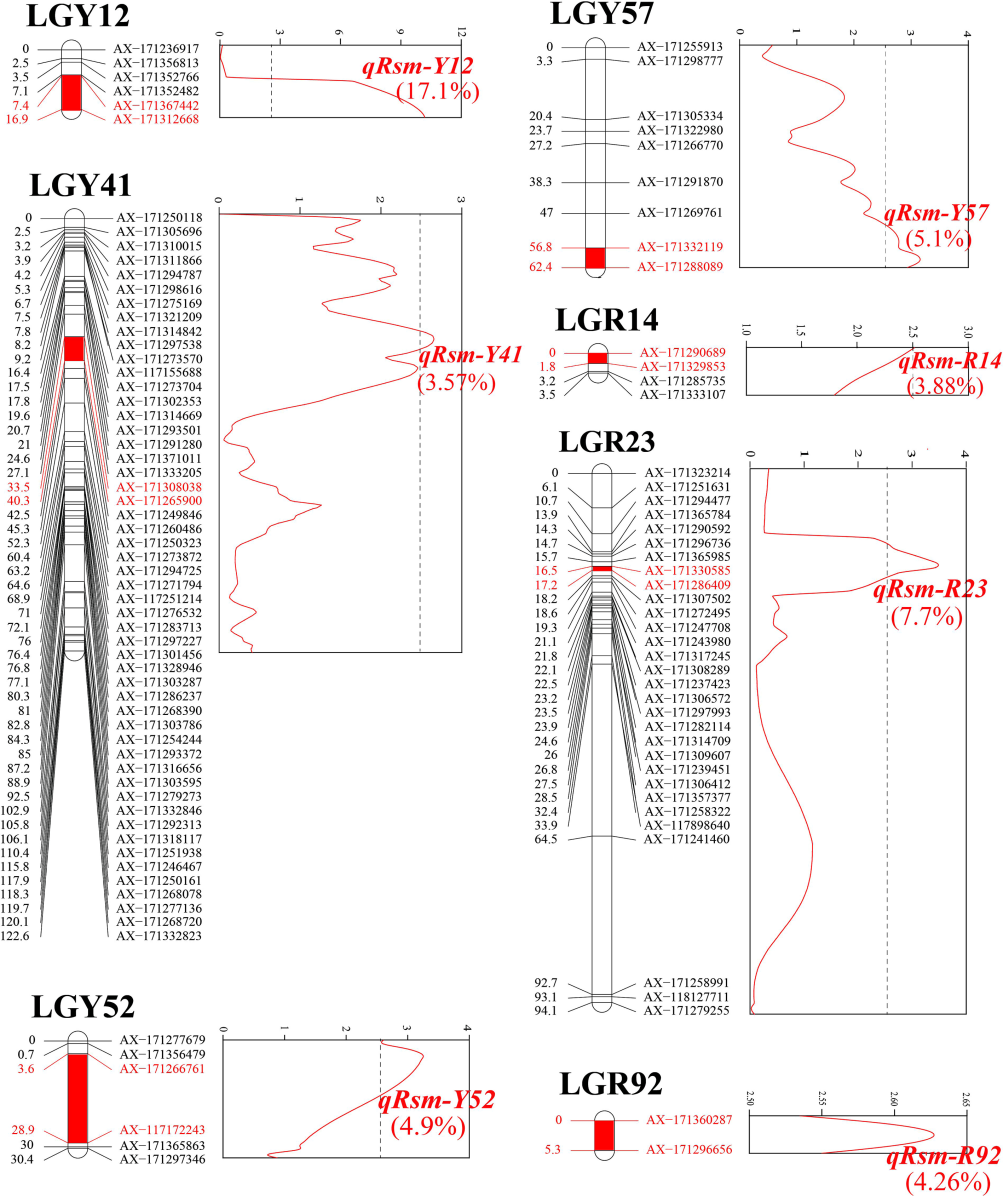
Location of seven SMD resistance-related QTLs (*q*) on sugarcane genetic linkage maps “Rsm”, resistance trait to sugarcane mosaic disease; “Y”, YT93-159; and “R”, ROC22. The colored text values, phenotypic variation explained (PVE).

Seven SMD resistance-related QTLs were detected (**Table 3**), which could explain 46.53% of the PVE. One major QTL, *qRsm-Y12*, could explain 17.10% of the PVE. The other six were minor QTLs, each could explain 3.57% ~ 7.70% of PVE. Four QTLs were detected on the YT93-159 map, and the remaining three QTLs were detected on the ROC22 map (**Figure 4**). The maximum genetic distance of each QTL from the nearest marker was 2.4 cM, the minimum was 0, and the average genetic distance was about 1.1 cM.

**Table 3:** SMD resistance-related QTLs in a F_1_ progeny mapping population from the YT93-159 × ROC22 cross.

QTL	Position	Left/Right markers	LOD	PVE(%)	Effect female	Effect male	Effect FM	GD (cM)	Marker^a^	Distance (cM)^b^
*qRsm-Y12*	16	AX-171367442/AX-171312668	10.19	17.10	-0.01	-0.05	0.50	9.5	AX-171312668	0.9
*qRsm-Y41*	35	AX-171308038/AX-171265900	2.72	3.57	0.06	-0.21	-0.11	6.8	AX-171308038	1.5
*qRsm-Y52*	4	AX-171266761/AX-117172243	3.25	4.90	0.27	-0.04	-0.02	25.3	AX-171266761	0.4
*qRsm-Y57*	60	AX-171332119/AX-171288089	3.37	5.12	0.19	-0.03	0.22	5.6	AX-171288089	2.4
*qRsm-R14*	0	AX-171290689/AX-171329853	2.52	3.88	-0.11	-0.10	0.19	1.8	AX-171290689	0
*qRsm-R23*	17	AX-171330585/AX-171286409	3.44	7.70	0.11	0.13	-0.25	0.7	AX-171286409	0.2
*qRsm-R92*	3	AX-171360287/AX-171296656	2.62	4.26	-0.22	0.12	-0.09	5.3	AX-171296656	2.3

“Y”, YT93-159; “R”, ROC22; LOD, logarithm of odds; PVE, phenotypic variation explained; GD, genetic distance between left and right markers; ^a^ Nearest marker from the QTL peak, ^b^ Distance of nearest marker from the respective QTL peak.

### Candidate gene mining

According to the sequence information of the markers on either side of the QTL (**Supplementary Table 11**), 1,525 candidate genes were searched in the seven QTLs regions. In total, 110 disease resistance candidate genes were identified, whose gene products included CC-TM (coiled-coil plus transmembrane receptor), LRR (leucine rich repeats), RLK (receptor-like protein kinases), WAK (wall-associated receptor kinase), and others domain. In addition, 69 transcription factors were identified, including AP2 (APETALA2), bHLH (basic helix-loop-helix), bZIP (basic region/leucine zipper), ERF (ethylene response factor), MYB (myeloblastosis), SBP (squamosa promoter binding protein) and other types of transcription factors (**Supplementary Table 12**). These genes and transcription factors may directly or indirectly involve in regulating sugarcane response to mosaic virus infection.

### Critical gene prediction

**Figure 5:**
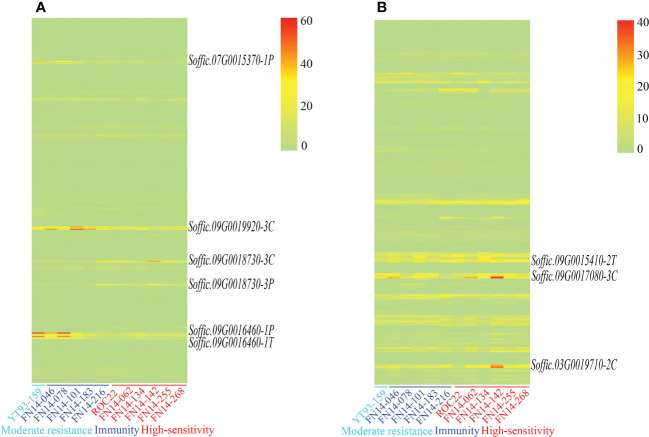
Expression of SMD resistance-related candidate genes **(A)** disease response-related genes; **(B)**. disease resistance-related transcription factors.

**Figure 6:**
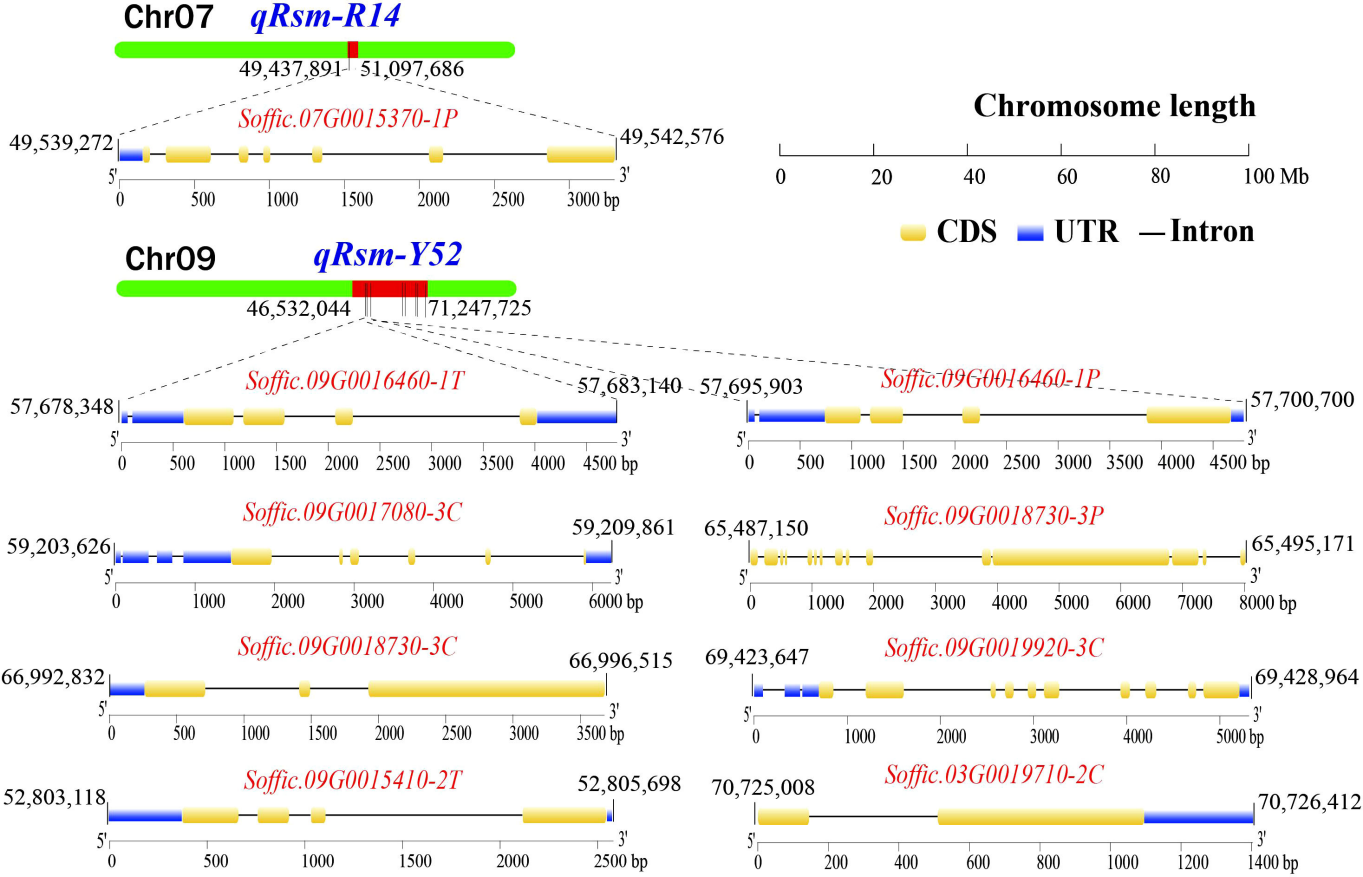
The genomic location, conserved domain, and gene structure of SMD resistance-related candidate key genes (UTR, untranslated region; CDS, coding sequence).

The gene expression levels of 110 pathogen-responsive genes and 69 transcription factors obtained by map mapping were presented in **Figure 5**. Among the candidate genes related to disease resistance, it was found that genes such as *Soffic.07G0015370-1P*, *Soffic.09G0016460-1T*, and *Soffic.09G0018730-3P* had significant expression differences between resistant and susceptible progenies, including three transcription factors and six pathogen response genes. These nine genes contained conserved domains such as bHLH_AtILR3_like, LRR, STKc_SNT7_plant and that were closely related to plant disease resistance (**Table 4**). The genomic positions, conserved domains and gene structures of the nine predicted genes are shown in **Figure 6**. It is speculated that these genes may be key to the resistance of sugarcane to SCMV and SrMV, and can be a focus for future research.

**Table 4:** Information for SMD resistance-related key genes.

No.	QTL	Candidate gene	*Arabidopsis* homologous gene	Conserved domain	Gene description
1	*qRsm-R14*	*Soffic.07G0015370-1P*	*AT2G43560*	FkpA super family	FKBP-like peptidyl-prolyl *cis-trans* isomerase family protein
2	*qRsm-Y52*	*Soffic.09G0015410-2T*	*AT5G54680*	bHLH_AtILR3_like	basic helix-loop-helix (bHLH) DNA-binding superfamily protein
3	*qRsm-Y52*	*Soffic.09G0016460-1T*	*AT5G01920*	STKc_SNT7_plant	Protein kinase superfamily protein
4	*qRsm-Y52*	*Soffic.09G0016460-1P*	*AT5G01920*	STKc_SNT7_plant	Protein kinase superfamily protein
5	*qRsm-Y52*	*Soffic.09G0017080-3C*	*AT3G12480*	BUR6 super family	nuclear factor Y, subunit C11
6	*qRsm-Y52*	*Soffic.09G0018730-3P*	*AT5G25930*	LRR	kinase family with leucine-rich repeat domain-containing protein
7	*qRsm-Y52*	*Soffic.09G0018730-3C*	*AT5G25930*	LRR	kinase family with leucine-rich repeat domain-containing protein
8	*qRsm-Y52*	*Soffic.09G0019920-3C*	*AT1G68830*	PLN03225	Serine/Threonine kinase domain protein
9	*qRsm-Y52*	*Soffic.03G0019710-2C*	*AT5G23000*	PLN03091 super family	myb domain protein 37

## Discussion

Mosaic disease is one of the most important viral diseases in sugarcane and has threatened the security and sustainability of the world sugarcane industry for a long time (Wu et al., 2012). In recent years, with the increasing pressure of natural stress, the differentiation of plant viruses has accelerated (Roossinck, 2015). The genetic basis of modern sugarcane cultivars is narrow, and the utilization of resistant genes and genotypes is limited. There is an increasing chance of a large-scale epidemic of mosaic diseases. Since different sugarcane varieties may have different resistances to the virus, breeding and careful distribution of disease-resistant varieties is the most economical and effective method to control mosaic disease. Therefore, it is imperative to fully explore the specifics of germplasm resistance and expand research on resistance-related molecular markers or key genes to further improve breeding efficiency.

In this study, SMD surveys were based on the “mosaic” symptom manifested under multiple environments. The results of resistance to mosaic disease in the experimental population showed that the overall disease incidence upon artificial inoculation was significantly higher than that upon natural infection. Due to many years of sugarcane production and greater levels of pathogen pressure, the overall disease incidence in sugarcane production areas of Guangxi and Yunnan is significantly higher than other ecological regions in China (**Supplementary Table 9**). In our study, inconsistent SMD incidences were observed across different habitats. The pathogen pressure of SMD was not high enough on the newly planted sugarcane crop at Cangshan ecological site (block 2) in 2020 and 2021, therefore, the survey data from these two environments were discarded. Therefore, the evaluation was only carried out with the progeny with the highest level of resistance across nine natural infection environments and three artificial inoculation infection environments. Excluding 29 F_1_ progeny with inconsistent levels of SMD resistance across different environments, 256 progeny of the F_1_ mapping population were included in further analysis. The 29 F_1_ progenies that were immune to SMD will be valuable in molecular breeding to develop SMD resistant sugarcane cultivars.

Sugarcane is a vegetatively propagated crop, and multiple sets of a mapping population can be propagated genetic research (Asnaghi et al., 2004). This study showed that the correlation coefficients among SMD resistance data sets from the various environments were highly significant (*p*< 0.001) at 0.26 ~ 0.91 (**Supplementary Table 10**). This indicates that SMD resistance is stable under different environmental conditions. The consolidated survey results showed that the frequency of the five grades followed a continuous normal distribution and that the Grades 1 and 2 contained 84 super-parent segregants with a better resistance level than the parent YT93-159, which is resistant to SMD (Grade 3) (**Figure 3**). This is in line with the typical characteristics of a quantitative trait controlled by polygenes. The generalized heritability (*H^2^*) of the SMD resistance across different environments was 0.85, which is obviously higher than the *H^2^* values reported on sugar content (0.57), plant height (0.57), effective stem number (0.65), single stem weight (0.56), and yield (0.49) (Barreto et al., 2019). This may be due to the long-term accumulation and habitation of the virus in sugarcane and the less effective management of SMD than on plant yield-related traits. The SMD resistance trait is mainly controlled by genetic factors, which can be identified using the map mapping method.

Mapping population size and molecular marker density directly affect the accuracy and resolution of marker localization for the target trait (Beavis, 1994). So far, most of the sugarcane populations for QTL mapping of agronomic traits are made up of between 100 and 200 individuals with traditional markers, such as AFLP, RFLP or SSR (Raboin et al., 2006; Yang, 2015; Singh et al., 2016). Due to the lack of detection tools, high-density genotyping of large populations, the genetic distance between the QTL markers and the gene of interest is relatively large (Daugrois et al., 1996; Raboin et al., 2006). In this study, linkage analysis was performed using a high-density map constructed by the Axiom Sugarcane 100K SNP chip, which contains 100,097 low-dose SNPs with a broad genetic basis and mainly distributed in gene regions. This chip includes 64,726 single-dose markers and 35,371 double-dose markers (You et al., 2019). Furthermore, the F_1_ progeny mapping population used in this study consisted of 256 eligible F_1_ progeny, which is significantly more than those of previous studies (Raboin et al., 2006; Yang et al., 2015; Singh et al., 2016).

The genetic analysis of SMD resistance was analyzed in this study. Seven SMD resistance-related QTLs were detected, only one of which, *qRsm-Y12*, was a major QTL that could explain 17.1% of the PVE. The genetic effect of *qRsm-Y12* is similar to the PVE effects seen for SCMV resistance (14.02%) by marker *AFLP-346* in sugarcane (Burbano et al., 2022) and the 15.3% ~ 15.8% PVE effect of a major QTL *R-scm3* related to SCMV resistance in maize (Zhang et al., 2003). The seven QTL markers identified in this study range in distance from the nearest marker from 0 to 2.4 cM, with an average of 1.1 cM, which is similar to those seen for sugarcane brown rust resistance-associated markers (0.1 cM ~ 8.1 cM) (Yang et al., 2017) and sugarcane orange rust markers (0.2 cM ~ 2.2 cM) (Yang et al., 2018). This further demonstrated the feasibility and reliability of using SNP genetic maps to locate target trait-related QTLs. However, even with a high-quality sugarcane SNP map, the distance of the closest markers on either side of the QTL is relatively large (Wang et al., 2021). For example, the distance between QTL *qRsm-Y57* and the closest marker is 2.4 cM, which makes target trait localization difficult and highlights the need for fine localization of SNP markers.

The major disease resistance traits in plants may generally be described by a gene-for-gene mechanism. The Avr products of pathogen-encoded avirulence genes are specifically recognized directly or indirectly by specific proteins encoded by the cognate plant disease resistance genes (Flor, 1971; Jia et al., 2000; Yakupjan et al., 2015). When plants sense a pathogen invasion signal, the disease resistance genes are activated through a series of signal transmissions. During this process, transcription factors play an important role in the defensive responses. For example, they may inhibit or activate the transcriptional expression of target genes by binding to specific DNA sequences in target gene promoters (Zhang et al., 2009). Plant leucine-rich repeat (LRR) receptor-like protein kinases represent a large group of protein families that play important roles in disease resistance (Smakowska-Luzan et al., 2018). Zhang et al. (2021) showed that a homologous *OsRLP1* gene regulated rice resistance to *Rice black-streaked dwarf virus* infection. Qi et al. (2014) found that a mutant of *A. thaliana* protein kinase AVRPPHB susceptible (PBS1) was defective in sensing the avirulence gene *avrPphB* of *Pseudomonas syringae*. Lee et al. (2015) showed that a serine/threonine kinase domain protein encoded by *OsPBL1* gene might play a role in rice stripe resistance. Chang et al. (2022) found a FKBP-type peptidyl-prolyl *cis-trans* isomerase (PPIase) could interact with the motor protein of *Tomato leaf curl New Delhi virus*, and its transient overexpression reduced the virus replication. Aparicio and Pallás (2017) confirmed that bHLH transcription factor can promote salicylic acid-dependent defense signaling by interacting with the *Alfalfa mosaic virus* CP protein. Studies have also shown that *MdMYB73* can improve apple’s resistance level to *Botryosphaeria dothidea* through the salicylic acid pathway (Gu et al., 2020). The expression of a MYB transcription factor *CaPHL8* was upregulated in *Ralstonia solanacerum* infected pepper plants. The upregulated expression activated the expressions of immune-related genes to enhance the defense response of pepper (Noman et al., 2019). O’Conner et al. (2021) showed that overexpression of *GmNF-YC4-2* in soybean increased seed protein content, exhibited a broad disease resistance, and accelerated soybean maturation.

In this study, a total of 110 pathogen-responsive genes and 69 transcription factors were identified in the interval regions of the QTLs. Among them, nine candidate genes were obtained in the interval region of the major QTL *qRsm-Y12*, including one transcription factor and eight resistance genes. Basically, plants share a common resistance mechanism to the same type of pathogen (Jones and Dangl, 2006; Li et al., 2020). SCMV and SrMV are the most widely distributed sugarcane mosaic virus in the world, with SCSMV mainly distributed in Asia (Lu et al., 2021). Therefore, we used an artificial inoculum that only contained SCMV and SrMV. Combined with the TPM normalization results of RNA-seq gene expression after inoculation of SCMV and SrMV, six genes and three transcription factors had significantly different levels of expression between resistant and susceptible materials. Two genes, *Soffic.09G0018730-3P* and *Soffic.09G0018730-3C*, contained LRR domains. Two genes, *Soffic.09G0016460-1T* and *Soffic.09G0016460-1P*, encoded kinase superfamily proteins. Gene *Soffic.09G0019920-3C* encoded a serine/threonine kinase domain protein. Gene *Soffic.07G0015370-1P* encoded a PPIase family protein. Among the transcription factors, *Soffic.09G0015410-2T* is a bHLH transcription factor, *Soffic.03G0019710-2C* encodes a MYB transcription factor, and *Soffic.09G0017080-3C* encodes a NF-YC transcription factor. It is thus speculated that these six genes and three transcription factors may have potential functions in sugarcane mosaic disease resistance.

## Conclusions

This study showed that the SMD resistance trait of 256 F_1_ progeny of a cross (YT93-159 × ROC22) tested under different environments was significantly correlated (*p*< 0.001) with correlation coefficients of 0.26~0.91, and hence was a highly heritable quantitative trait (*H^2^* = 0.85). Based on the consolidated multiple data sets of SMD resistance, 29 immune, 55 highly resistant, 70 moderately resistant, 62 susceptible, and 40 highly susceptible F_1_ progeny were identified. Using a high-quality SNP chip, seven SMD resistance-related QTLs were located. One major QTL, *qRsm-Y12*, explained 17.10% of the PVE and six minor QTLs, namely, *qRsm-Y41*, *qRsm-Y52*, *qRsm-Y57*, *qRsm-R14*, *qRsm-R23*, and *qRsm-R92*, explained 3.57% ~ 7.70% of the PVE. A total of 110 SMD response genes and 69 transcription factors were screened for association with SMD resistance. Six key genes, namely, *Soffic.07G0015370-1P*, *Soffic.09G0016460-1T*, *Soffic.09G0016460-1P*, *Soffic.09G0018730-3P*, *Soffic.09G0018730-3C*, and *Soffic.09G0019920-3C* and three transcription factors, namely, *Soffic.09G0015410-2T*, *Soffic.09G0017080-3C*, and *Soffic.03G0019710-2C*, were identified. These genes and transcription factors can be further explored and utilized in the marker-assisted breeding for mosaic disease resistance in sugarcane.

## Data availability statement

The original contributions presented in the study are publicly available. This data can be found here: https://www.ncbi.nlm.nih.gov/, PRJNA918436.

## Author contributions

GL, YQ and LX conceptualized the study. GL, YQ and LX designed the experiments. GL, Y-BP and YQ prepared the manuscript draft. GL, Y-BP, LX, and YQ reviewed and edited the manuscript. LX provided the materials. GL, ZW, QW, WC, FX, SD and BL performed the experiments. GL and ZW conducted the data analysis. All authors contributed to the article and approved the submitted version.
