# Supplementary material for: Identification of QTLs and critical genes related to sugarcane mosaic disease resistance
Source: Front Plant Sci. 2023 Feb 2;14:1107314. doi: 10.3389/fpls.2023.1107314 (PMC9932707; doi:10.3389/fpls.2023.1107314)
Supplement: Supplementary file 1 [file DataSheet_1.docx]

Supplementary Material


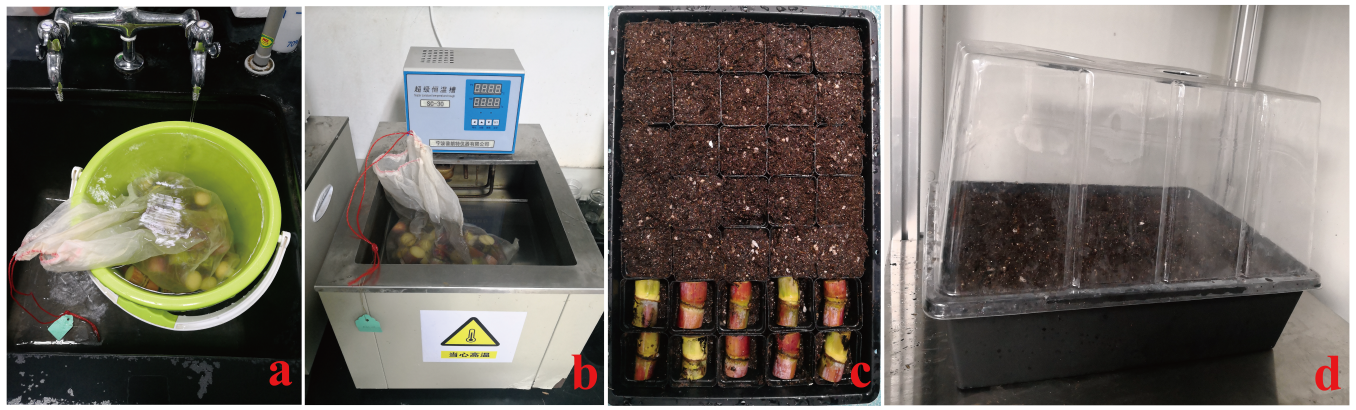


**Figure S1.** Virus-free seedlings from cane stems

*a.* *running water treatment; b. detoxification in a constant temperature water bath; c. cultivation with sterilized substrate; d. conservation culture.*


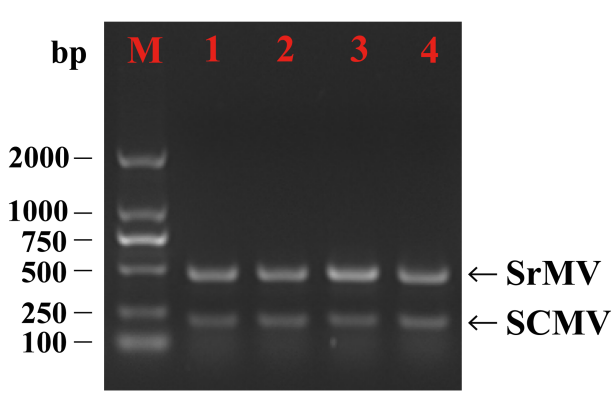


**Figure S2.** RT-PCR products of mixed virus solutions

*M: DNA Marker 2000, 1: YT93-159 and ROC22 inoculated with mixed virus solution; 2-4: the first, second, and third progeny population inoculation with mixed virus solution, respectively.*


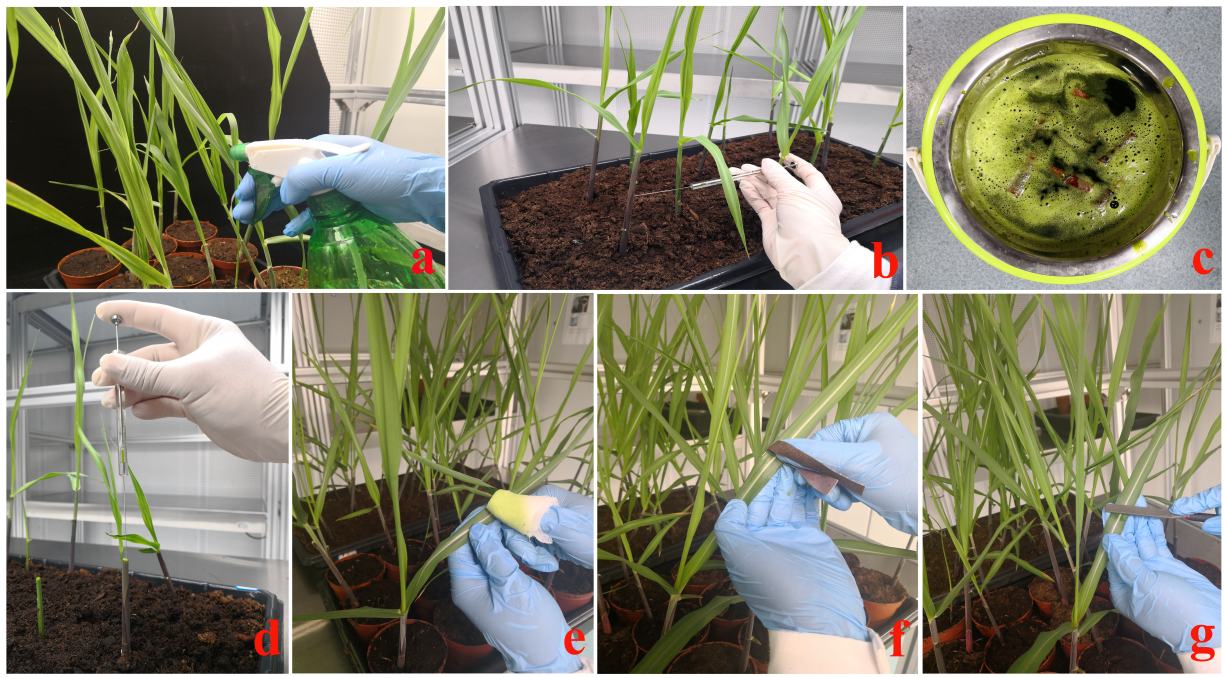


**Figure S3.** Schematic diagrams of different inoculation methods

1. *spray inoculation; b. micro-injection; c. single bud soaking; d. young stem cut inoculation; e. quartz sand friction; f. abrasive cloth friction; and g. rasp friction.*

**
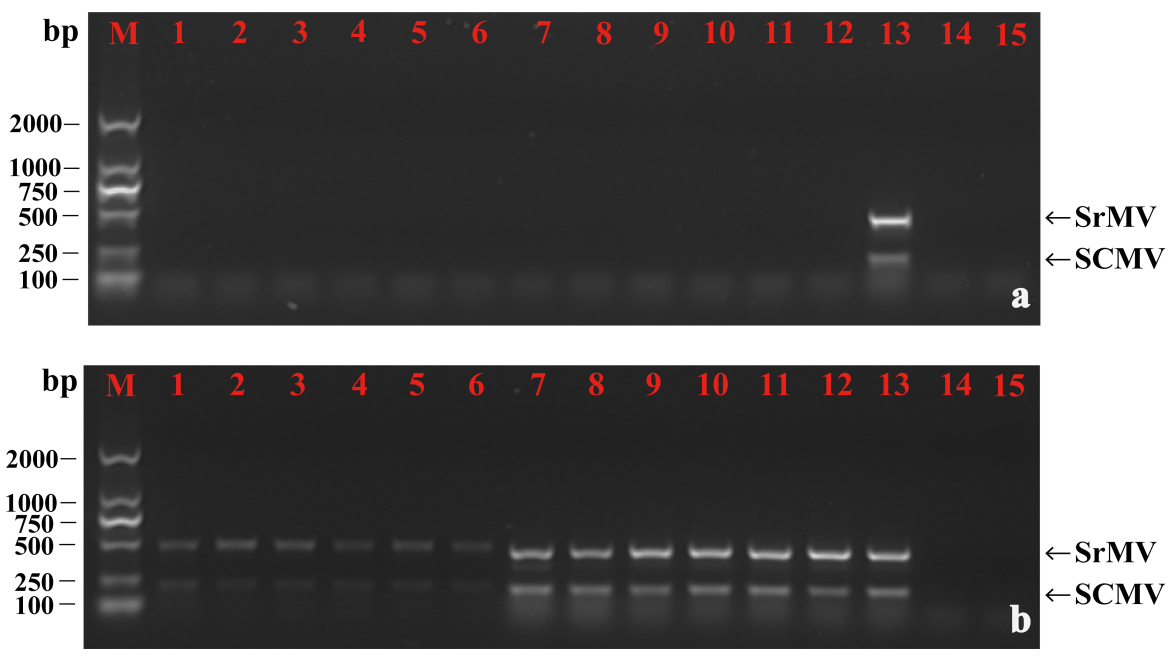
**

**Figure S4.** RT-PCR products from hot water-detoxified seedlings (a) and four days after inoculation with mosaic viruses (b) of YT93-159, ROC22, and 10 F_1_ progenies

*Lane M: DNA Marker 2000; lanes 1: FN14-46; 2: FN14-78; 3: FN14-101; 4: FN14-183; 5: FN14-216; 6: YT93-159; 7: ROC22; 8: FN14- 62; 9: FN14-134; 10: FN14-142; 11: FN14-255; 12: FN14-268; 13: positive control; 14: negative control; and 15: blank control*.


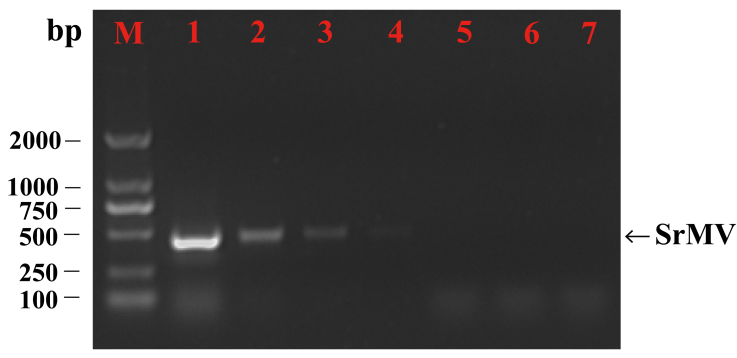


**Figure S5.** RT-PCR products from plantlets derived from hot water detoxified buds of FN14-255

*M: DNA Marker 2000, 1: positive control (CK); 2-6: treated at 50°C, 55°C, 57°C, 59°C and 61°C, respectively; 7: negative control.*


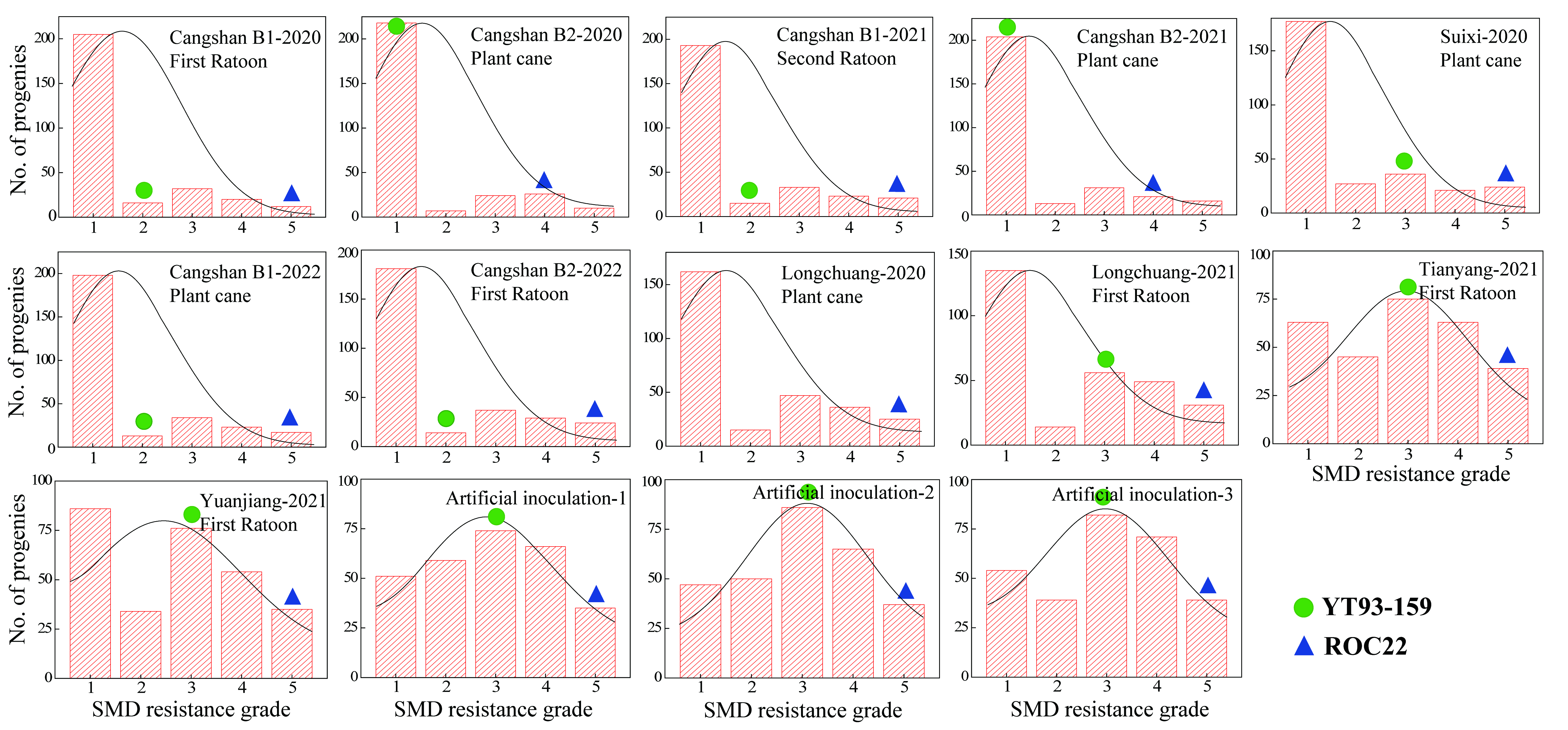


**Figure S6.** Frequency distribution of SMD response in a F_1_ population under 14 environments

**Table S1.** Geographical characteristics of ecological regions for testing the F_1_ progeny population

| **No.** | **Location** | **Longitude and latitude** | **Altitude (m)** | **Perennial average temperature (℃)** | **Perennial average precipitation (mm)** | **Climate type** |
| --- | --- | --- | --- | --- | --- | --- |
| 1 | Cangshan, Fujian | 119˚14’E  26˚5’N | 85 | 20.1 | 1375 | Subtropical marine monsoon climate |
| 2 | Longchuan, Yunnan | 97˚53’E  24˚15’N | 718 | 18.8 | 1618 | South-Asia tropical monsoon climate |
| 3 | Suixi, Guangdong | 110˚10’E  21˚6’N | 22 | 23.2 | 1802 | Subtropical marine monsoon climate |
| 4 | Tianyang, Guangxi | 107˚0’E  23˚39’N | 113 | 22.0 | 1100 | South-Asia tropical monsoon climate |
| 5 | Yuanjiang, Yunnan | 101˚59’E  23˚36’N | 620 | 23.8 | 788 | Subtropical valley climate |

**Table S2.** Planting density of the F_1_ populations tested under different ecological regions

| **No.** | **Region** | **Row length (m)** | **Row spacing (m)** | **Number of buds/m** |
| --- | --- | --- | --- | --- |
| 1 | Cangshan, Fujian | 1.0 | 1.2 | 14 |
| 2 | Longchuan, Yunnan | 1.0 | 1.0 | 14 |
| 3 | Suixi, Guangdong | 5.0 | 1.2 | 10 |
| 4 | Tianyang, Guangxi | 3.0 | 1.2 | 12 |
| 5 | Yuanjiang, Yunnan | 3.0 | 1.0 | 14 |

**Table S3.** Description of eight artificial inoculation methods

| **No.** | **Inoculation method** | **Description** | **Reference** |
| --- | --- | --- | --- |
| 1 | Spray inoculation | use a high-pressure watering nozzle to spray the virus liquid evenly on the young leaves | Dean, 1960 |
| 2 | Micro-inject inoculation | inject a quantitative amount of venom into young stems with a micro-syringe | Zhou, 2015 |
| 3 | Quartz sand friction | friction inoculation, with fingers dipped in a little virus-containing liquid and quartz sand to artificially scratch and infect young leaves | Li et al., 2013 |
| 4 | Abrasive cloth friction | use 1.0 cm wide sandpaper (120#) dipped in a little virus solution and gently rub the leaves in the same direction for 5 times | - |
| 5 | Rasp friction | use a rasp with a width of 1.0 cm to dip a little virus liquid and gently rub the leaves in the same direction for 5 times | - |
| 6 | Cut young stems | cut young stem 3~5 cm away from the ground with a sterile scalpel, use a pipette gun to drop 50 µL virus solution on the wound, and wrap the wound with absorbent cotton | Li et al., 2018 |
| 7 | Single bud soaking | mature stem was cut into single buds (about 5 cm in length) and immersed in the inoculated venom for 1.0 min, no detoxification was performed in this method | - |
| 8 | Single bud soaking + quartz sand friction | on the basis of single bud soaking, quartz sand friction inoculation was started when the cane had grown to 2~3 leaves | - |

**Table S4.** Information related to the genetic map of YT93-159 and ROC22

| **Parent** | **Number of linkage groups** | **Total length (cM)** | **Number of markers** | **Average marker density (cM/marker)** |
| --- | --- | --- | --- | --- |
| YT93-159 | 93 | 4,485.2 | 1,497 | 3.0 |
| ROC22 | 92 | 2,720.0 | 776 | 3.5 |

**Table S5.** The quality of the RNA samples from this study

| **No.** | **Sample name** | **Nucleic acid number** | **Concentration (µg/µl)** | **Volume (µl)** | **Total (µg)** | **Integrity value** | **Quality** |
| --- | --- | --- | --- | --- | --- | --- | --- |
| 1 | YT-0d-1 | FKRN220008061-1A | 752 | 42 | 31.58 | 7.2 | A |
| 2 | YT-0d-2 | FKRN220008062-1A | 616 | 43 | 26.49 | 6.6 | A |
| 3 | YT-0d-3 | FKRN220008134-1A | 632 | 42 | 26.54 | 7.5 | A |
| 4 | YT-1d-1 | FKRN220008086-1A | 1,002 | 41 | 41.08 | 7.7 | A |
| 5 | YT-1d-2 | FKRN220008109-1A | 933 | 16 | 14.93 | 7.9 | A |
| 6 | YT-1d-3 | FKRN220008157-1A | 1,383 | 41 | 56.70 | 7.0 | A |
| 7 | YT-4d-1 | FKRN220008107-1A | 891 | 46 | 40.99 | 7.9 | A |
| 8 | YT-4d-2 | FKRN220008108-1A | 1,162 | 48 | 55.78 | 7.9 | A |
| 9 | YT-4d-3 | FKRN220008110-1A | 522 | 26 | 13.57 | 7.8 | A |
| 10 | ROC22-0d-1 | FKRN220008131-1A | 589 | 41 | 24.15 | 7.9 | A |
| 11 | ROC22-0d-2 | FKRN220008132-1A | 739 | 40 | 29.56 | 7.9 | A |
| 12 | ROC22-0d-3 | FKRN220008133-1A | 541 | 41 | 22.18 | 7.0 | A |
| 13 | ROC22-1d-1 | FKRN220008155-1A | 1,432 | 44 | 63.01 | 7.1 | A |
| 14 | ROC22-1d-2 | FKRN220008156-1A | 1,265 | 41 | 51.87 | 7.4 | A |
| 15 | ROC22-1d-3 | FKRN220008158-1A | 1,352 | 42 | 56.78 | 6.7 | A |
| 16 | ROC22-4d-1 | FKRN220008179-1A | 1,304 | 39 | 50.86 | 7.4 | A |
| 17 | ROC22-4d-2 | FKRN220008180-1A | 2,330 | 32 | 74.56 | 7.2 | A |
| 18 | ROC22-4d-3 | FKRN220008182-1A | 1,284 | 27 | 34.67 | 7.2 | A |
| 19 | 046-0d-1 | FKRN220008064-1A | 756 | 45 | 34.02 | 7.2 | A |
| 20 | 046-0d-2 | FKRN220008065-1A | 705 | 44 | 31.02 | 7.6 | A |
| 21 | 046-0d-3 | FKRN220008066-1A | 722 | 42 | 30.32 | 7.5 | A |
| 22 | 046-1d-1 | FKRN220008087-1A | 655 | 39 | 25.55 | 6.8 | A |
| 23 | 046-1d-2 | FKRN220008089-1A | 456 | 40 | 18.24 | 6.5 | A |
| 24 | 046-1d-3 | FKRN220008090-1A | 642 | 19 | 12.20 | 7.6 | A |
| 25 | 046-4d-1 | FKRN220008112-1A | 340 | 46 | 15.64 | 6.9 | A |
| 26 | 046-4d-2 | FKRN220008113-1A | 418 | 30 | 12.54 | 7.1 | A |
| 27 | 046-4d-3 | FKRN220008114-1A | 649 | 29 | 18.82 | 7.8 | A |
| 28 | 062-0d-1 | FKRN220008135-1A | 973 | 42 | 40.87 | 7.7 | A |

Continued table S5.

| **No.** | **Sample name** | **Nucleic acid number** | **Concentration (ng/µl)** | **Volume (µl)** | **Total (µg)** | **Integrity value** | **Quality** |
| --- | --- | --- | --- | --- | --- | --- | --- |
| 29 | 062-0d-2 | FKRN220008136-1A | 997 | 43 | 42.87 | 7.4 | A |
| 30 | 062-0d-3 | FKRN220008138-1A | 944 | 41 | 38.70 | 6.6 | A |
| 31 | 062-1d-1 | FKRN220008159-1A | 1,264 | 42 | 53.09 | 6.9 | A |
| 32 | 062-1d-2 | FKRN220008160-1A | 1,370 | 42 | 57.54 | 7.1 | A |
| 33 | 062-1d-3 | FKRN220008162-1A | 1,218 | 40 | 48.72 | 7.8 | A |
| 34 | 062-4d-1 | FKRN220008183-1A | 1,295 | 31 | 40.15 | 7.2 | A |
| 35 | 062-4d-2 | FKRN220008161-1A | 1,062 | 42 | 44.60 | 7.8 | A |
| 36 | 062-4d-3 | FKRN220008186-1A | 1,030 | 27 | 27.81 | 6.9 | A |
| 37 | 078-0d-1 | FKRN220008067-1A | 835 | 48 | 40.08 | 7.9 | A |
| 38 | 078-0d-2 | FKRN220008068-1A | 1,068 | 46 | 49.13 | 7.2 | A |
| 39 | 078-0d-3 | FKRN220008069-1A | 1,076 | 45 | 48.42 | 6.9 | A |
| 40 | 078-1d-1 | FKRN220008091-1A | 1,062 | 33 | 35.05 | 6.5 | A |
| 41 | 078-1d-2 | FKRN220008093-1A | 859 | 30 | 25.77 | 6.3 | A |
| 42 | 078-1d-3 | FKRN220008094-1A | 920 | 32 | 29.44 | 7.1 | A |
| 43 | 078-4d-1 | FKRN220008115-1A | 544 | 46 | 25.02 | 7.2 | A |
| 44 | 078-4d-2 | FKRN220008116-1A | 707 | 41 | 28.99 | 7.7 | A |
| 45 | 078-4d-3 | FKRN220008118-1A | 886 | 30 | 26.58 | 7.9 | A |
| 46 | 101-0d-1 | FKRN220008071-1A | 874 | 44 | 38.46 | 6.5 | A |
| 47 | 101-0d-2 | FKRN220008072-1A | 691 | 40 | 27.64 | 6.6 | A |
| 48 | 101-0d-3 | FKRN220008074-1A | 801 | 45 | 36.05 | 7.6 | A |
| 49 | 101-1d-1 | FKRN220008073-1A | 765 | 45 | 34.43 | 7.3 | A |
| 50 | 101-1d-2 | FKRN220008097-1A | 624 | 35 | 21.84 | 7.2 | A |
| 51 | 101-1d-3 | FKRN220008098-1A | 765 | 31 | 23.72 | 7.4 | A |
| 52 | 101-4d-1 | FKRN220008119-1A | 875 | 46 | 40.25 | 7.7 | A |
| 53 | 101-4d-2 | FKRN220008120-1A | 875 | 44 | 38.50 | 7.9 | A |
| 54 | 101-4d-3 | FKRN220008121-1A | 1,035 | 29 | 30.02 | 7.1 | A |
| 55 | 134-0d-1 | FKRN220008141-1A | 727 | 42 | 30.53 | 7.1 | A |
| 56 | 134-0d-2 | FKRN220008142-1A | 469 | 40 | 18.76 | 7.0 | A |
| 57 | 134-0d-3 | FKRN220008181-1A | 1,341 | 33 | 44.25 | 6.8 | A |
| 58 | 134-1d-1 | FKRN220008163-1A | 487 | 40 | 19.48 | 7.8 | A |

Continued table S5.

| **No.** | **Sample name** | **Nucleic acid number** | **Concentration (ng/µl)** | **Volume (µl)** | **Total (µg)** | **Integrity value** | **Quality** |
| --- | --- | --- | --- | --- | --- | --- | --- |
| 59 | 134-1d-2 | FKRN220008164-1A | 948 | 34 | 32.23 | 7.3 | A |
| 60 | 134-1d-3 | FKRN220008190-1A | 724 | 25 | 18.10 | 7.0 | A |
| 61 | 134-4d-1 | FKRN220008187-1A | 637 | 46 | 29.30 | 7.3 | A |
| 62 | 134-4d-2 | FKRN220008188-1A | 782 | 46 | 35.97 | 7.3 | A |
| 63 | 134-4d-3 | FKRN220008189-1A | 553 | 27 | 14.93 | 6.9 | A |
| 64 | 142-0d-1 | FKRN220008144-1A | 1,610 | 41 | 66.01 | 6.8 | A |
| 65 | 142-0d-2 | FKRN220008143-1A | 736 | 41 | 30.18 | 7.1 | A |
| 66 | 142-0d-3 | FKRN220008146-1A | 1,332 | 44 | 58.61 | 6.9 | A |
| 67 | 142-1d-1 | FKRN220008167-1A | 1,112 | 21 | 23.35 | 6.5 | A |
| 68 | 142-1d-2 | FKRN220008168-1A | 1,468 | 24 | 35.23 | 6.6 | A |
| 69 | 142-1d-3 | FKRN220008170-1A | 1,135 | 21 | 23.84 | 6.3 | A |
| 70 | 142-4d-1 | FKRN220008191-1A | 862 | 27 | 23.27 | 6.8 | A |
| 71 | 142-4d-2 | FKRN220008192-1A | 1,211 | 42 | 50.86 | 7.7 | A |
| 72 | 142-4d-3 | FKRN220008194-1A | 1,180 | 28 | 33.04 | 6.5 | A |
| 73 | 183-0d-1 | FKRN220008075-1A | 575 | 40 | 23.00 | 6.3 | A |
| 74 | 183-0d-2 | FKRN220008077-1A | 600 | 50 | 30.00 | 6.4 | A |
| 75 | 183-0d-3 | FKRN220008126-1A | 585 | 29 | 16.97 | 7.5 | A |
| 76 | 183-1d-1 | FKRN220008099-1A | 521 | 30 | 15.63 | 6.1 | A |
| 77 | 183-1d-2 | FKRN220008100-1A | 378 | 33 | 12.47 | 6.3 | A |
| 78 | 183-1d-3 | FKRN220008102-1A | 381 | 30 | 11.43 | 6.2 | A |
| 79 | 183-4d-1 | FKRN220008123-1A | 1,127 | 42 | 47.33 | 7.2 | A |
| 80 | 183-4d-2 | FKRN220008124-1A | 1,362 | 46 | 62.65 | 7.5 | A |
| 81 | 183-4d-3 | FKRN220008125-1A | 679 | 29 | 19.69 | 6.8 | A |
| 82 | 216-0d-1 | FKRN220008079-1A | 643 | 46 | 29.58 | 7.9 | A |
| 83 | 216-0d-2 | FKRN220008080-1A | 293 | 45 | 13.19 | 7.5 | A |
| 84 | 216-0d-3 | FKRN220008081-1A | 639 | 42 | 26.84 | 7.8 | A |
| 85 | 216-1d-1 | FKRN220008103-1A | 989 | 38 | 37.58 | 8.3 | A |
| 86 | 216-1d-2 | FKRN220008104-1A | 995 | 53 | 52.74 | 8.2 | A |
| 87 | 216-1d-3 | FKRN220008105-1A | 931 | 41 | 38.17 | 8.6 | A |

Continued table S5.

| **No.** | **Sample name** | **Nucleic acid number** | **Concentration (ng/µl)** | **Volume (µl)** | **Total (µg)** | **Integrity value** | **Quality** |
| --- | --- | --- | --- | --- | --- | --- | --- |
| 88 | 216-4d-1 | FKRN220008127-1A | 823 | 39 | 32.10 | 8.3 | A |
| 89 | 216-4d-2 | FKRN220008128-1A | 812 | 39 | 31.67 | 8.4 | A |
| 90 | 216-4d-3 | FKRN220008129-1A | 1,018 | 25 | 25.45 | 8.1 | A |
| 91 | 255-0d-1 | FKRN220008148-1A | 1,080 | 42 | 45.36 | 6.5 | A |
| 92 | 255-0d-2 | FKRN220008150-1A | 1,316 | 43 | 56.59 | 6.8 | A |
| 93 | 255-0d-3 | FKRN220008106-1A | 834 | 37 | 30.86 | 7.2 | A |
| 94 | 255-1d-1 | FKRN220008171-1A | 424 | 34 | 14.42 | 6.5 | A |
| 95 | 255-1d-2 | FKRN220008130-1A | 996 | 25 | 24.90 | 8.4 | A |
| 96 | 255-1d-3 | FKRN220008145-1A | 1,054 | 41 | 43.21 | 6.5 | A |
| 97 | 255-4d-1 | FKRN220008195-1A | 2,228 | 41 | 91.35 | 6.7 | A |
| 98 | 255-4d-2 | FKRN220008197-1A | 1,514 | 27 | 40.88 | 6.6 | A |
| 99 | 255-4d-3 | FKRN220008198-1A | 1,333 | 30 | 39.99 | 6.9 | A |
| 100 | 268-0d-1 | FKRN220008151-1A | 1,088 | 43 | 46.78 | 6.9 | A |
| 101 | 268-0d-2 | FKRN220008152-1A | 1,249 | 44 | 54.96 | 6.9 | A |
| 102 | 268-0d-3 | FKRN220008154-1A | 1,174 | 44 | 51.66 | 7.1 | A |
| 103 | 268-1d-1 | FKRN220008175-1A | 1,374 | 42 | 57.71 | 7.2 | A |
| 104 | 268-1d-2 | FKRN220008176-1A | 1,094 | 37 | 40.48 | 6.6 | A |
| 105 | 268-1d-3 | FKRN220008178-1A | 720 | 26 | 18.72 | 7.7 | A |
| 106 | 268-4d-1 | FKRN220008199-1A | 885 | 39 | 34.52 | 7.2 | A |
| 107 | 268-4d-2 | FKRN220008200-1A | 873 | 41 | 35.79 | 7.7 | A |
| 108 | 268-4d-3 | FKRN220008202-1A | 1,234 | 26 | 32.08 | 7.3 | A |

**Table S6.** The quality of transcriptome sequencing data

| **Sample name** | **Library** | **Raw reads** | **Clean reads** | **Raw base(G)** | **Clean base(G)** | **Effective rate(%)** | **Error rate(%)** | **Phred > 20**  **Q20(%)** | **Phred > 30**  **Q30(%)** | **GC content(%)** |
| --- | --- | --- | --- | --- | --- | --- | --- | --- | --- | --- |
| YT-0d-1 | FRAS220008061-4a | 51,813,871 | 50,125,897 | 15.54 | 15.04 | 96.74 | 0.03 | 97.46 | 92.32 | 61.61 |
| YT-0d-2 | FRAS220008061-3a | 61,514,980 | 59,378,886 | 18.45 | 17.81 | 96.53 | 0.03 | 97.18 | 91.52 | 61.42 |
| YT-0d-3 | FRAS220008062-3a | 64,170,691 | 62,576,483 | 19.25 | 18.77 | 97.52 | 0.03 | 97.41 | 92.19 | 61.93 |
| YT-1d-1 | FRAS220008086-5a | 68,642,345 | 66,573,884 | 20.59 | 19.97 | 96.99 | 0.03 | 97.88 | 93.60 | 58.98 |
| YT-1d-2 | FRAS220008086-6a | 64,008,086 | 62,210,906 | 19.20 | 18.66 | 97.19 | 0.03 | 97.69 | 93.03 | 59.04 |
| YT-1d-3 | FRAS220008086-7a | 56,987,996 | 55,724,381 | 17.10 | 16.72 | 97.78 | 0.03 | 97.63 | 92.83 | 58.91 |
| YT-4d-1 | FRAS220008107-2a | 58,408,536 | 56,741,661 | 17.52 | 17.02 | 97.15 | 0.03 | 97.61 | 92.75 | 59.74 |
| YT-4d-2 | FRAS220008108-2a | 42,124,216 | 40,758,565 | 12.64 | 12.23 | 96.76 | 0.03 | 97.43 | 92.18 | 60.01 |
| YT-4d-3 | FRAS220008109-2a | 59,822,259 | 58,120,928 | 17.95 | 17.44 | 97.16 | 0.03 | 97.53 | 92.56 | 60.74 |
| ROC22-0d-1 | FRAS220008131-2a | 88,451,254 | 86,002,489 | 26.54 | 25.80 | 97.23 | 0.03 | 97.50 | 92.53 | 60.27 |
| ROC22-0d-2 | FRAS220008132-2a | 78,587,186 | 76,256,760 | 23.58 | 22.88 | 97.03 | 0.03 | 97.74 | 93.22 | 60.30 |
| ROC22-0d-3 | FRAS220008134-1a | 44,811,682 | 43,720,922 | 13.44 | 13.12 | 97.57 | 0.03 | 97.29 | 92.41 | 60.08 |
| ROC22-1d-1 | FRAS220008155-3a | 67,166,894 | 65,635,971 | 20.15 | 19.69 | 97.72 | 0.03 | 97.43 | 92.26 | 58.81 |
| ROC22-1d-2 | FRAS220008156-3a | 32,923,288 | 31,967,726 | 9.88 | 9.59 | 97.10 | 0.03 | 97.36 | 92.02 | 58.68 |
| ROC22-1d-3 | FRAS220008157-3a | 56,805,840 | 55,402,439 | 17.04 | 16.62 | 97.53 | 0.03 | 97.70 | 93.01 | 57.95 |
| ROC22-4d-1 | FRAS220008179-3a | 68,769,761 | 66,523,149 | 20.63 | 19.96 | 96.73 | 0.03 | 97.43 | 92.25 | 59.91 |
| ROC22-4d-2 | FRAS220008181-1a | 54,163,398 | 52,862,714 | 16.25 | 15.86 | 97.60 | 0.03 | 97.88 | 93.85 | 57.22 |

Continued table S6.

| **Sample name** | **Library** | **Raw reads** | **Clean reads** | **Raw base(G)** | **Clean base(G)** | **Effective rate(%)** | **Error rate(%)** | **Phred > 20**  **Q20(%)** | **Phred > 30**  **Q30(%)** | **GC content(%)** |
| --- | --- | --- | --- | --- | --- | --- | --- | --- | --- | --- |
| ROC22-4d-3 | FRAS220008182-3a | 59,836,473 | 57,798,961 | 17.95 | 17.34 | 96.59 | 0.03 | 97.64 | 92.77 | 58.33 |
| 046-0d-1 | FRAS220008064-2a | 66,166,414 | 63,393,013 | 19.85 | 19.02 | 95.81 | 0.03 | 97.91 | 93.62 | 60.94 |
| 046-0d-2 | FRAS220008066-4a | 58,332,819 | 56,710,978 | 17.50 | 17.01 | 97.22 | 0.03 | 97.75 | 93.20 | 60.22 |
| 046-0d-3 | FRAS220008066-3a | 56,366,462 | 54,722,201 | 16.91 | 16.42 | 97.08 | 0.03 | 97.78 | 93.31 | 60.31 |
| 046-1d-1 | FRAS220008087-5a | 53,456,451 | 51,860,488 | 16.04 | 15.56 | 97.01 | 0.03 | 97.44 | 92.18 | 59.75 |
| 046-1d-2 | FRAS220008087-4a | 61,036,479 | 59,633,481 | 18.31 | 17.89 | 97.70 | 0.03 | 97.76 | 93.25 | 59.77 |
| 046-1d-3 | FRAS220008089-2a | 64,314,427 | 62,582,006 | 19.29 | 18.77 | 97.31 | 0.03 | 97.73 | 93.15 | 59.48 |
| 046-4d-1 | FRAS220008112-2a | 61,282,877 | 59,774,154 | 18.38 | 17.93 | 97.54 | 0.03 | 97.73 | 93.06 | 60.61 |
| 046-4d-2 | FRAS220008113-3a | 74,106,544 | 71,945,597 | 22.23 | 21.58 | 97.08 | 0.03 | 96.38 | 90.50 | 61.76 |
| 046-4d-3 | FRAS220008114-2a | 64,193,559 | 62,009,661 | 19.26 | 18.60 | 96.60 | 0.03 | 98.06 | 94.09 | 60.41 |
| 062-0d-1 | FRAS220008135-3a | 61,336,097 | 59,606,026 | 18.40 | 17.88 | 97.18 | 0.03 | 97.59 | 92.86 | 61.09 |
| 062-0d-2 | FRAS220008136-4a | 61,150,578 | 58,960,556 | 18.35 | 17.69 | 96.42 | 0.03 | 97.17 | 91.63 | 61.26 |
| 062-0d-3 | FRAS220008138-3a | 61,789,387 | 59,641,473 | 18.54 | 17.89 | 96.52 | 0.03 | 97.57 | 92.75 | 60.59 |
| 062-1d-1 | FRAS220008160-3a | 57,671,491 | 55,898,759 | 17.30 | 16.77 | 96.93 | 0.03 | 97.50 | 92.50 | 59.40 |
| 062-1d-2 | FRAS220008161-3a | 55,547,090 | 540,169,73 | 16.66 | 16.21 | 97.25 | 0.03 | 97.45 | 92.34 | 59.73 |
| 062-1d-3 | FRAS220008162-3a | 70,255,416 | 68,609,703 | 21.08 | 20.58 | 97.66 | 0.03 | 97.91 | 93.69 | 59.80 |
| 062-4d-1 | FRAS220008183-5a | 65,999,697 | 64,038,678 | 19.80 | 19.21 | 97.03 | 0.03 | 97.78 | 93.25 | 59.98 |

Continued table S6.

| **Sample name** | **Library** | **Raw reads** | **Clean reads** | **Raw base(G)** | **Clean base(G)** | **Effective rate(%)** | **Error rate(%)** | **Phred > 20**  **Q20(%)** | **Phred > 30**  **Q30(%)** | **GC content(%)** |
| --- | --- | --- | --- | --- | --- | --- | --- | --- | --- | --- |
| 062-4d-2 | FRAS220008183-4a | 73,031,810 | 70,745,796 | 21.91 | 21.22 | 96.87 | 0.03 | 97.52 | 92.51 | 59.80 |
| 062-4d-3 | FRAS220008186-3a | 72,149,188 | 70,251,171 | 21.64 | 21.08 | 97.37 | 0.03 | 97.67 | 92.90 | 60.19 |
| 078-0d-1 | FRAS220008067-2a | 56,233,303 | 54,465,678 | 16.87 | 16.34 | 96.86 | 0.03 | 98.02 | 93.98 | 61.32 |
| 078-0d-2 | FRAS220008068-2a | 58,764,073 | 57,210,577 | 17.63 | 17.16 | 97.36 | 0.03 | 97.70 | 93.06 | 61.36 |
| 078-0d-3 | FRAS220008069-2a | 61,524,114 | 59,294,660 | 18.46 | 17.79 | 96.38 | 0.03 | 97.84 | 93.40 | 60.79 |
| 078-1d-1 | FRAS220008091-2a | 57,611,731 | 55,901,539 | 17.28 | 16.77 | 97.03 | 0.03 | 97.87 | 93.59 | 59.97 |
| 078-1d-2 | FRAS220008093-1a | 40,911,643 | 39,822,683 | 12.27 | 11.95 | 97.34 | 0.03 | 96.75 | 90.97 | 60.77 |
| 078-1d-3 | FRAS220008094-3a | 58,700,531 | 56,978,711 | 17.61 | 17.09 | 97.07 | 0.03 | 97.16 | 91.52 | 59.87 |
| 078-4d-1 | FRAS220008117-2a | 64,379,540 | 62,328,794 | 19.31 | 18.70 | 96.81 | 0.03 | 97.72 | 93.06 | 60.58 |
| 078-4d-2 | FRAS220008118-2a | 57,201,416 | 55,452,120 | 17.16 | 16.64 | 96.94 | 0.03 | 97.82 | 93.37 | 60.10 |
| 078-4d-3 | FRAS220008116-2a | 59,665,646 | 57,928,820 | 17.90 | 17.38 | 97.09 | 0.03 | 97.84 | 93.44 | 61.56 |
| 101-0d-1 | FRAS220008072-2a | 61,482,032 | 59,556,427 | 18.44 | 17.87 | 96.87 | 0.03 | 97.51 | 92.49 | 60.84 |
| 101-0d-2 | FRAS220008073-2a | 60,844,319 | 59,041,008 | 18.25 | 17.71 | 97.04 | 0.03 | 97.89 | 93.62 | 60.33 |
| 101-0d-3 | FRAS220008074-2a | 60,470,833 | 58,428,576 | 18.14 | 17.53 | 96.62 | 0.03 | 97.75 | 93.22 | 61.14 |
| 101-1d-1 | FRAS220008096-3a | 68,579,182 | 66,100,950 | 20.57 | 19.83 | 96.39 | 0.03 | 96.34 | 90.39 | 59.87 |
| 101-1d-2 | FRAS220008097-2a | 68,032,355 | 65,973,522 | 20.41 | 19.79 | 96.97 | 0.03 | 97.77 | 93.19 | 58.30 |
| 101-1d-3 | FRAS220008098-1a | 59,573,141 | 57,694,926 | 17.87 | 17.31 | 96.85 | 0.03 | 96.98 | 91.59 | 60.71 |

Continued table S6.

| **Sample name** | **Library** | **Raw reads** | **Clean reads** | **Raw base(G)** | **Clean base(G)** | **Effective rate(%)** | **Error rate(%)** | **Phred > 20**  **Q20(%)** | **Phred > 30**  **Q30(%)** | **GC content(%)** |
| --- | --- | --- | --- | --- | --- | --- | --- | --- | --- | --- |
| 101-4d-1 | FRAS220008119-2a | 65,140,281 | 63,193,973 | 19.54 | 18.96 | 97.01 | 0.03 | 97.67 | 92.96 | 60.79 |
| 101-4d-2 | FRAS220008120-1a | 110,151,942 | 108,202,306 | 33.05 | 32.46 | 98.23 | 0.03 | 97.89 | 93.84 | 59.51 |
| 101-4d-3 | FRAS220008122-1a | 40,575,860 | 39,919,166 | 12.17 | 11.98 | 98.38 | 0.03 | 97.19 | 92.05 | 60.13 |
| 134-0d-1 | FRAS220008141-4a | 68,508,877 | 66,557,472 | 20.55 | 19.97 | 97.15 | 0.03 | 97.73 | 93.10 | 60.33 |
| 134-0d-2 | FRAS220008141-5a | 58,191,269 | 56,386,223 | 17.46 | 16.92 | 96.90 | 0.03 | 97.69 | 93.02 | 60.23 |
| 134-0d-3 | FRAS220008142-3a | 57,042,337 | 54,454,246 | 17.11 | 16.34 | 95.46 | 0.03 | 98.16 | 94.30 | 56.34 |
| 134-1d-1 | FRAS220008163-4a | 62,668,982 | 61,282,009 | 18.80 | 18.38 | 97.79 | 0.03 | 97.23 | 91.55 | 59.87 |
| 134-1d-2 | FRAS220008163-5a | 63,990,321 | 62,281,234 | 19.20 | 18.68 | 97.33 | 0.03 | 97.70 | 93.06 | 59.98 |
| 134-1d-3 | FRAS220008164-3a | 56,846,561 | 54,794,545 | 17.05 | 16.44 | 96.39 | 0.03 | 97.81 | 93.34 | 59.34 |
| 134-4d-1 | FRAS220008187-3a | 69,783,446 | 67,280,041 | 20.94 | 20.18 | 96.41 | 0.03 | 97.78 | 93.23 | 59.91 |
| 134-4d-2 | FRAS220008188-3a | 60,791,103 | 58,135,729 | 18.24 | 17.44 | 95.63 | 0.03 | 97.55 | 92.59 | 60.68 |
| 134-4d-3 | FRAS220008190-3a | 64,454,595 | 62,064,670 | 19.34 | 18.62 | 96.29 | 0.03 | 97.84 | 93.44 | 60.38 |
| 142-0d-1 | FRAS220008143-3a | 48,472,793 | 46,875,536 | 14.54 | 14.06 | 96.70 | 0.03 | 97.94 | 93.78 | 59.89 |
| 142-0d-2 | FRAS220008144-3a | 57,013,946 | 55,209,589 | 17.10 | 16.56 | 96.84 | 0.03 | 97.93 | 93.81 | 60.65 |
| 142-0d-3 | FRAS220008146-3a | 57,579,190 | 55,775,477 | 17.27 | 16.73 | 96.87 | 0.03 | 97.71 | 93.18 | 60.42 |
| 142-1d-1 | FRAS220008167-2a | 36,393,558 | 35,213,026 | 10.92 | 10.56 | 96.76 | 0.03 | 96.17 | 89.56 | 60.98 |
| 142-1d-2 | FRAS220008168-3a | 57,158,747 | 55,240,787 | 17.15 | 16.57 | 96.64 | 0.03 | 97.56 | 92.69 | 60.58 |

Continued table S6.

| **Sample name** | **Library** | **Raw reads** | **Clean reads** | **Raw base(G)** | **Clean base(G)** | **Effective rate(%)** | **Error rate(%)** | **Phred > 20**  **Q20(%)** | **Phred > 30**  **Q30(%)** | **GC content(%)** |
| --- | --- | --- | --- | --- | --- | --- | --- | --- | --- | --- |
| 142-1d-3 | FRAS220008170-3a | 56,414,282 | 54,393,279 | 16.92 | 16.32 | 96.42 | 0.03 | 97.44 | 92.34 | 61.26 |
| 142-4d-1 | FRAS220008191-3a | 68,254,892 | 65,629,263 | 20.48 | 19.69 | 96.15 | 0.03 | 97.84 | 93.45 | 61.70 |
| 142-4d-2 | FRAS220008192-3a | 58,373,289 | 56,406,360 | 17.51 | 16.92 | 96.63 | 0.03 | 97.81 | 93.37 | 60.74 |
| 142-4d-3 | FRAS220008194-4a | 69,723,911 | 66,581,718 | 20.92 | 19.97 | 95.49 | 0.03 | 97.85 | 93.50 | 60.95 |
| 183-0d-1 | FRAS220008075-2a | 64,709,476 | 62,891,113 | 19.41 | 18.87 | 97.19 | 0.03 | 97.82 | 93.46 | 60.77 |
| 183-0d-2 | FRAS220008077-3a | 48,763,421 | 47,467,875 | 14.63 | 14.24 | 97.34 | 0.03 | 97.47 | 92.48 | 61.16 |
| 183-0d-3 | FRAS220008077-4a | 37,894,634 | 36,911,023 | 11.37 | 11.07 | 97.40 | 0.03 | 97.59 | 92.84 | 61.09 |
| 183-1d-1 | FRAS220008099-1a | 53,029,722 | 51,526,009 | 15.91 | 15.46 | 97.16 | 0.03 | 97.06 | 91.80 | 60.13 |
| 183-1d-2 | FRAS220008100-1a | 52,460,547 | 50,994,792 | 15.74 | 15.30 | 97.21 | 0.03 | 96.34 | 90.02 | 60.67 |
| 183-1d-3 | FRAS220008101-2a | 68,436,622 | 66,465,224 | 20.53 | 19.94 | 97.12 | 0.03 | 98.00 | 93.97 | 58.98 |
| 183-4d-1 | FRAS220008123-2a | 62,520,736 | 61,025,217 | 18.76 | 18.31 | 97.61 | 0.03 | 97.87 | 93.45 | 59.79 |
| 183-4d-2 | FRAS220008124-2a | 58,244,026 | 56,609,720 | 17.47 | 16.98 | 97.19 | 0.03 | 98.21 | 94.53 | 59.32 |
| 183-4d-3 | FRAS220008126-2a | 34,002,891 | 33,072,941 | 10.20 | 9.92 | 97.27 | 0.03 | 97.41 | 92.31 | 61.24 |
| 216-0d-1 | FRAS220008079-2a | 57,495,556 | 55,875,665 | 17.25 | 16.76 | 97.18 | 0.03 | 97.62 | 92.80 | 60.10 |
| 216-0d-2 | FRAS220008080-2a | 64,783,669 | 62,640,073 | 19.44 | 18.79 | 96.69 | 0.03 | 97.75 | 93.19 | 58.31 |
| 216-0d-3 | FRAS220008081-2a | 70,668,613 | 68,780,207 | 21.20 | 20.63 | 97.33 | 0.03 | 97.50 | 92.34 | 59.97 |
| 216-1d-1 | FRAS220008103-1a | 50,778,149 | 49,723,981 | 15.23 | 14.92 | 97.92 | 0.03 | 97.89 | 93.71 | 57.48 |

Continued table S6.

| **Sample name** | **Library** | **Raw reads** | **Clean reads** | **Raw base(G)** | **Clean base(G)** | **Effective rate(%)** | **Error rate(%)** | **Phred > 20**  **Q20(%)** | **Phred > 30**  **Q30(%)** | **GC content(%)** |
| --- | --- | --- | --- | --- | --- | --- | --- | --- | --- | --- |
| 216-1d-2 | FRAS220008104-2a | 72,437,427 | 70,455,241 | 21.73 | 21.14 | 97.26 | 0.03 | 97.94 | 93.81 | 58.83 |
| 216-1d-3 | FRAS220008105-2a | 66,396,617 | 64,503,683 | 19.92 | 19.35 | 97.15 | 0.03 | 97.77 | 93.25 | 58.53 |
| 216-4d-1 | FRAS220008127-2a | 59,453,874 | 57,714,767 | 17.84 | 17.31 | 97.07 | 0.03 | 97.95 | 93.77 | 59.15 |
| 216-4d-2 | FRAS220008128-2a | 75,005,181 | 73,126,596 | 22.50 | 21.94 | 97.50 | 0.03 | 97.98 | 93.83 | 58.49 |
| 216-4d-3 | FRAS220008130-2a | 70,701,179 | 68,559,793 | 21.21 | 20.57 | 96.97 | 0.03 | 98.14 | 94.33 | 59.29 |
| 255-0d-1 | FRAS220008148-3a | 70,488,419 | 68,504,207 | 21.15 | 20.55 | 97.19 | 0.03 | 97.56 | 92.68 | 61.12 |
| 255-0d-2 | FRAS220008150-4a | 54,687,000 | 53,052,999 | 16.41 | 15.92 | 97.01 | 0.03 | 97.75 | 93.19 | 60.05 |
| 255-0d-3 | FRAS220008150-5a | 58,867,785 | 56,857,152 | 17.66 | 17.06 | 96.58 | 0.03 | 97.52 | 92.51 | 60.13 |
| 255-1d-1 | FRAS220008171-2a | 55,740,919 | 54,644,844 | 16.72 | 16.39 | 98.03 | 0.03 | 97.37 | 92.60 | 59.89 |
| 255-1d-2 | FRAS220008171-1a | 59,350,119 | 58,117,962 | 17.81 | 17.44 | 97.92 | 0.03 | 96.94 | 91.44 | 59.88 |
| 255-1d-3 | FRAS220008171-3a | 54,656,848 | 53,696,515 | 16.40 | 16.11 | 98.24 | 0.03 | 97.06 | 91.80 | 59.93 |
| 255-4d-1 | FRAS220008195-3a | 65,335,443 | 63,262,539 | 19.60 | 18.98 | 96.83 | 0.03 | 97.91 | 93.66 | 60.11 |
| 255-4d-2 | FRAS220008197-3a | 59,540,762 | 57,328,420 | 17.86 | 17.20 | 96.28 | 0.03 | 97.42 | 92.24 | 59.53 |
| 255-4d-3 | FRAS220008198-4a | 65,060,171 | 62,781,647 | 19.52 | 18.83 | 96.50 | 0.03 | 97.72 | 93.16 | 61.87 |
| 268-0d-1 | FRAS220008151-3a | 55,593,096 | 54,382,374 | 16.68 | 16.31 | 97.82 | 0.03 | 97.43 | 92.31 | 61.67 |
| 268-0d-2 | FRAS220008152-3a | 60,353,034 | 59,002,933 | 18.11 | 17.70 | 97.76 | 0.03 | 97.39 | 92.16 | 61.86 |
| 268-0d-3 | FRAS220008154-3a | 60,893,807 | 59,575,268 | 18.27 | 17.87 | 97.83 | 0.03 | 97.66 | 92.98 | 62.33 |

Continued table S6.

| **Sample name** | **Library** | **Raw reads** | **Clean reads** | **Raw base(G)** | **Clean base(G)** | **Effective rate(%)** | **Error rate(%)** | **Phred > 20**  **Q20(%)** | **Phred > 30**  **Q30(%)** | **GC content(%)** |
| --- | --- | --- | --- | --- | --- | --- | --- | --- | --- | --- |
| 268-1d-1 | FRAS220008175-3a | 54,863,570 | 53,406,323 | 16.46 | 16.02 | 97.34 | 0.03 | 97.84 | 93.53 | 60.05 |
| 268-1d-2 | FRAS220008176-3a | 57,805,812 | 55,830,264 | 17.34 | 16.75 | 96.58 | 0.03 | 97.48 | 92.47 | 60.47 |
| 268-1d-3 | FRAS220008178-3a | 66,036,777 | 64,249,353 | 19.81 | 19.27 | 97.29 | 0.03 | 97.46 | 92.41 | 60.89 |
| 268-4d-1 | FRAS220008199-3a | 73,600,264 | 71,016,695 | 22.08 | 21.31 | 96.49 | 0.03 | 97.73 | 93.09 | 61.12 |
| 268-4d-2 | FRAS220008201-3a | 61,366,843 | 58,831,712 | 18.41 | 17.65 | 95.87 | 0.03 | 97.85 | 93.46 | 59.79 |
| 268-4d-3 | FRAS220008202-3a | 48,539,823 | 46,423,455 | 14.56 | 13.93 | 95.64 | 0.03 | 97.97 | 93.82 | 60.36 |

**Table S7.** Germination rates of sugarcane buds treated by different hot water temperatures

| **Treatment temperature (℃)** | **Germination rates after planting (%)** | | |
| --- | --- | --- | --- |
|  | **10 days** | **20 days** | **30 days** |
| CK | 93.33 ± 3.34 a | 100.00 ± 0.00 a | 100.00 ± 0.00 a |
| 55 | 35.55 ± 6.94 b | 55.56 ± 3.85 b | 70.00 ± 6.67 b |
| 57 | 16.67 ± 3.33 c | 45.56 ± 5.09 c | 55.56 ± 5.09 c |
| 59 | 10.00 ± 3.31 c | 21.11 ± 5.09 d | 31.11 ± 5.09 d |
| 61 | 0.00 d | 6.67 ± 3.34 e | 8.89 ± 3.84 e |

*Note: Data were presented by mean ± standard deviation. Different lowercase letters within the same column indicate significant difference among different treatments (P < 0.05).*

**Table S8.** Inoculation efficiency of different inoculation methods

| **Inoculation method** | | **First survey (%)** | | | **Second survey (%)** | | | **Third survey (%)** | |
| --- | --- | --- | --- | --- | --- | --- | --- | --- | --- |
|  |  | **YT93-159** | **ROC22** |  | **YT93-159** | **ROC22** |  | **YT93-159** | **ROC22** |
| Spray | | 14.81 ± 3.70 cd | 26.19 ± 5.45 c | | 16.04 ± 2.14 cd | 28.57 ± 7.14 ef | | 17.28 ± 2.14 c | 28.57 ± 7.14 e |
| Micro-inject | | 5.66 ± 1.93 ef | 14.94 ± 1.99 d | | 5.56 ± 1.93 e | 16.09 ± 1.99 g | | 5.56 ± 1.93 e | 16.09 ± 1.99 f |
| Friction | Quartz sand friction | 22.22 ± 5.09 ab | 64.37 ± 7.18 a | | 23.33 ± 3.34 ab | 68.97 ± 6.90 b | | 23.33 ± 3.34 ab | 68.97 ± 6.90 b |
|  | Abrasive cloth friction | 11.50 ± 1.99 de | 36.90 ± 5.46 b | | 12.64 ± 1.99 d | 36.90 ± 5.46 de | | 12.64 ± 1.99 d | 36.90 ± 5.46 de |
|  | Rasp friction | 17.86 ± 3.57 bcd | 40.23 ± 7.18 b | | 16.67 ± 2.06 cd | 41.38 ± 9.12 cd | | 17.86 ± 3.57 c | 41.38 ± 9.12 cd |
| Stalk cutting | Cut young stems | 2.78 ± 2.41 f | 16.66 ± 2.22 cd | | 4.17 ± 4.17 e | 16.66 ± 2.22 g | | 5.56 ± 2.40 e | 17.95 ± 2.22 f |
|  | Single bud soaking | 19.44 ± 4.19 abc | 46.67 ± 8.82 b | | 20.00 ± 3.33 bc | 50.00 ± 6.67 c | | 20.00 ± 3.33 bc | 52.22 ± 7.70 c |
| Single bud soaking + quartz sand friction | | 23.47 ± 1.85 a | 73.56 ± 5.27 a | | 24.81 ± 3.29 a | 79.31 ± 3.45 a | | 27.19 ± 1.92 a | 82.76 ± 6.90 a |

*Note: The first, second, and third surveys were conducted in 2W, 3W, and 4W, respectively. Data were presented by mean ± standard deviation. Different lowercase letters within the same column indicate a significant difference (P < 0.05).*

**Table S9.** SMD survey data of YT93-159, ROC22, and the F_1_ progeny population

| **Variety (Line)** | **2020** | | | **2021** | | | | | | **2022** | | **Artificial inoculation** | | | **Resistance level** |
| --- | --- | --- | --- | --- | --- | --- | --- | --- | --- | --- | --- | --- | --- | --- | --- |
|  | **CS-b1** | **CS-b2** | **LC** | **CS-b1** | **CS-b2** | **LC** | **YJ** | **TY** | **SX** | **CS-b1** | **CS-b2** | **No.1** | **No.2** | **No.3** |  |
| YT93-159 | 2 | 1 | 3 | 2 | 1 | 3 | 3 | 3 | 3 | 2 | 2 | 3 | 3 | 3 | 3 |
| ROC22 | 5 | 4 | 5 | 5 | 4 | 5 | 5 | 5 | 5 | 5 | 5 | 5 | 5 | 5 | 5 |
| 9 | 1 | 1 | 1 | 1 | 1 | 1 | 1 | 1 | 1 | 1 | 1 | 1 | 1 | 1 | 1 |
| 13 | 1 | 1 | 1 | 1 | 1 | 1 | 1 | 1 | 1 | 1 | 1 | 1 | 1 | 1 | 1 |
| 15 | 1 | 1 | 1 | 1 | 1 | 1 | 1 | 1 | 1 | 1 | 1 | 1 | 1 | 1 | 1 |
| 25 | 1 | 1 | 1 | 1 | 1 | 1 | 1 | 1 | 1 | 1 | 1 | 1 | 1 | 1 | 1 |
| 46 | 1 | 1 | 1 | 1 | 1 | 1 | 1 | 1 | 1 | 1 | 1 | 1 | 1 | 1 | 1 |
| 66 | 1 | 1 | 1 | 1 | 1 | 1 | 1 | 1 | 1 | 1 | 1 | 1 | 1 | 1 | 1 |
| 73 | 1 | 1 | 1 | 1 | 1 | 1 | 1 | 1 | 1 | 1 | 1 | 1 | 1 | 1 | 1 |
| 78 | 1 | 1 | 1 | 1 | 1 | 1 | 1 | 1 | 1 | 1 | 1 | 1 | 1 | 1 | 1 |
| 101 | 1 | 1 | 1 | 1 | 1 | 1 | 1 | 1 | 1 | 1 | 1 | 1 | 1 | 1 | 1 |
| 102 | 1 | 1 | 1 | 1 | 1 | 1 | 1 | 1 | 1 | 1 | 1 | 1 | 1 | 1 | 1 |
| 118 | 1 | 1 | 1 | 1 | 1 | 1 | 1 | 1 | 1 | 1 | 1 | 1 | 1 | 1 | 1 |
| 140 | 1 | 1 | 1 | 1 | 1 | 1 | 1 | 1 | 1 | 1 | 1 | 1 | 1 | 1 | 1 |
| 141 | 1 | 1 | 1 | 1 | 1 | 1 | 1 | 1 | 1 | 1 | 1 | 1 | 1 | 1 | 1 |
| 156 | 1 | 1 | 1 | 1 | 1 | 1 | 1 | 1 | 1 | 1 | 1 | 1 | 1 | 1 | 1 |
| 158 | 1 | 1 | 1 | 1 | 1 | 1 | 1 | 1 | 1 | 1 | 1 | 1 | 1 | 1 | 1 |
| 163 | 1 | 1 | 1 | 1 | 1 | 1 | 1 | 1 | 1 | 1 | 1 | 1 | 1 | 1 | 1 |
| 169 | 1 | 1 | 1 | 1 | 1 | 1 | 1 | 1 | 1 | 1 | 1 | 1 | 1 | 1 | 1 |
| 182 | 1 | 1 | 1 | 1 | 1 | 1 | 1 | 1 | 1 | 1 | 1 | 1 | 1 | 1 | 1 |
| 183 | 1 | 1 | 1 | 1 | 1 | 1 | 1 | 1 | 1 | 1 | 1 | 1 | 1 | 1 | 1 |
| 186 | 1 | 1 | 1 | 1 | 1 | 1 | 1 | 1 | 1 | 1 | 1 | 1 | 1 | 1 | 1 |
| 208 | 1 | 1 | 1 | 1 | 1 | 1 | 1 | 1 | 1 | 1 | 1 | 1 | 1 | 1 | 1 |
| 216 | 1 | 1 | 1 | 1 | 1 | 1 | 1 | 1 | 1 | 1 | 1 | 1 | 1 | 1 | 1 |
| 250 | 1 | 1 | 1 | 1 | 1 | 1 | 1 | 1 | 1 | 1 | 1 | 1 | 1 | 1 | 1 |
| 259 | 1 | 1 | 1 | 1 | 1 | 1 | 1 | 1 | 1 | 1 | 1 | 1 | 1 | 1 | 1 |
| 284 | 1 | 1 | 1 | 1 | 1 | 1 | 1 | 1 | 1 | 1 | 1 | 1 | 1 | 1 | 1 |
| 347 | 1 | 1 | 1 | 1 | 1 | 1 | 1 | 1 | 1 | 1 | 1 | 1 | 1 | 1 | 1 |
| 355 | 1 | 1 | 1 | 1 | 1 | 1 | 1 | 1 | 1 | 1 | 1 | 1 | 1 | 1 | 1 |
| 371 | 1 | 1 | 1 | 1 | 1 | 1 | 1 | 1 | 1 | 1 | 1 | 1 | 1 | 1 | 1 |
| 405 | 1 | 1 | 1 | 1 | 1 | 1 | 1 | 1 | 1 | 1 | 1 | 1 | 1 | 1 | 1 |

Continued table S9.

| **Variety (Line)** | **2020** | | | **2021** | | | | | | **2022** | | **Artificial inoculation** | | | **Resistance level** |
| --- | --- | --- | --- | --- | --- | --- | --- | --- | --- | --- | --- | --- | --- | --- | --- |
|  | **CS-b1** | **CS-b2** | **LC** | **CS-b1** | **CS-b2** | **LC** | **YJ** | **TY** | **SX** | **CS-b1** | **CS-b2** | **No.1** | **No.2** | **No.3** |  |
| 8 | 1 | 1 | 1 | 1 | 2 | 1 | 1 | 2 | 2 | 1 | 1 | 2 | 2 | 2 | 2 |
| 19 | 1 | 1 | 1 | 1 | 1 | 1 | 1 | 2 | 1 | 1 | 1 | 2 | 2 | 2 | 2 |
| 30 | 1 | 1 | 1 | 1 | 1 | 1 | 1 | 2 | 1 | 1 | 1 | 2 | 2 | 2 | 2 |
| 34 | 1 | 1 | 1 | 1 | 1 | 1 | 1 | 2 | 1 | 1 | 1 | 2 | 1 | 2 | 2 |
| 39 | 1 | 1 | 2 | 1 | 1 | 2 | 2 | 2 | 1 | 1 | 1 | 1 | 2 | 2 | 2 |
| 41 | 1 | 1 | 1 | 1 | 1 | 1 | 1 | 2 | 1 | 1 | 1 | 2 | 2 | 2 | 2 |
| 42 | 1 | 1 | 2 | 1 | 1 | 1 | 1 | 2 | 1 | 1 | 1 | 2 | 1 | 2 | 2 |
| 48 | 1 | 1 | 1 | 1 | 1 | 2 | 2 | 2 | 1 | 1 | 1 | 2 | 2 | 2 | 2 |
| 51 | 1 | 1 | 1 | 1 | 1 | 1 | 1 | 1 | 2 | 1 | 1 | 2 | 2 | 2 | 2 |
| 65 | 1 | 1 | 1 | 2 | 1 | 1 | 1 | 1 | 1 | 1 | 1 | 2 | 2 | 2 | 2 |
| 67 | 1 | 1 | 1 | 1 | 1 | 2 | 2 | 2 | 2 | 1 | 1 | 2 | 2 | 2 | 2 |
| 96 | 1 | 1 | 1 | 1 | 1 | 1 | 2 | 2 | 1 | 1 | 1 | 2 | 1 | 2 | 2 |
| 109 | 1 | 1 | 1 | 1 | 1 | 1 | 2 | 2 | 1 | 1 | 1 | 2 | 2 | 2 | 2 |
| 114 | 1 | 1 | 1 | 1 | 1 | 1 | 1 | 2 | 2 | 1 | 1 | 2 | 2 | 2 | 2 |
| 127 | 1 | 1 | 1 | 1 | 1 | 1 | 2 | 2 | 1 | 1 | 1 | 2 | 1 | 2 | 2 |
| 131 | 1 | 1 | 1 | 1 | 1 | 1 | 2 | 2 | 1 | 2 | 1 | 2 | 2 | 2 | 2 |
| 144 | 1 | 1 | 1 | 1 | 1 | 1 | 2 | 1 | 1 | 2 | 1 | 2 | 2 | 2 | 2 |
| 147 | 1 | 1 | 1 | 2 | 1 | 1 | 1 | 1 | 1 | 2 | 1 | 2 | 2 | 2 | 2 |
| 148 | 1 | 1 | 1 | 1 | 1 | 1 | 2 | 1 | 1 | 1 | 1 | 2 | 2 | 2 | 2 |
| 151 | 1 | 1 | 1 | 1 | 1 | 1 | 1 | 1 | 1 | 1 | 1 | 2 | 2 | 2 | 2 |
| 157 | 1 | 1 | 1 | 1 | 2 | 1 | 1 | 1 | 1 | 1 | 1 | 2 | 2 | 2 | 2 |
| 175 | 1 | 1 | 1 | 1 | 1 | 1 | 2 | 2 | 1 | 1 | 1 | 2 | 2 | 2 | 2 |
| 188 | 1 | 1 | 1 | 1 | 1 | 1 | 2 | 2 | 1 | 1 | 1 | 2 | 1 | 2 | 2 |
| 189 | 1 | 1 | 1 | 1 | 2 | 1 | 2 | 2 | 1 | 1 | 1 | 2 | 2 | 2 | 2 |
| 197 | 1 | 1 | 1 | 1 | 1 | 1 | 2 | 2 | 1 | 1 | 1 | 2 | 2 | 2 | 2 |
| 205 | 1 | 1 | 1 | 1 | 2 | 1 | 2 | 2 | 1 | 1 | 1 | 2 | 2 | 2 | 2 |
| 206 | 1 | 1 | 1 | 1 | 1 | 1 | 2 | 2 | 1 | 1 | 1 | 2 | 2 | 2 | 2 |
| 215 | 1 | 1 | 1 | 1 | 1 | 1 | 2 | 2 | 2 | 1 | 1 | 2 | 2 | 2 | 2 |
| 217 | 1 | 1 | 1 | 1 | 1 | 2 | 1 | 1 | 1 | 1 | 1 | 2 | 2 | 2 | 2 |
| 219 | 1 | 1 | 1 | 2 | 1 | 1 | 1 | 1 | 1 | 1 | 1 | 2 | 1 | 2 | 2 |
| 221 | 1 | 1 | 1 | 1 | 1 | 1 | 2 | 2 | 1 | 1 | 1 | 2 | 2 | 2 | 2 |

Continued table S9.

| **Variety (Line)** | **2020** | | | **2021** | | | | | | **2022** | | **Artificial inoculation** | | | **Resistance level** |
| --- | --- | --- | --- | --- | --- | --- | --- | --- | --- | --- | --- | --- | --- | --- | --- |
|  | **CS-b1** | **CS-b2** | **LC** | **CS-b1** | **CS-b2** | **LC** | **YJ** | **TY** | **SX** | **CS-b1** | **CS-b2** | **No.1** | **No.2** | **No.3** |  |
| 225 | 1 | 1 | 1 | 1 | 1 | 1 | 2 | 2 | 2 | 1 | 1 | 2 | 2 | 2 | 2 |
| 227 | 1 | 1 | 2 | 2 | 1 | 2 | 1 | 1 | 2 | 1 | 1 | 2 | 2 | 2 | 2 |
| 232 | 1 | 1 | 1 | 1 | 1 | 1 | 2 | 2 | 1 | 1 | 1 | 2 | 2 | 2 | 2 |
| 234 | 1 | 1 | 1 | 2 | 1 | 1 | 1 | 1 | 1 | 1 | 1 | 2 | 2 | 2 | 2 |
| 235 | 1 | 1 | 1 | 1 | 1 | 1 | 2 | 2 | 1 | 1 | 1 | 2 | 2 | 2 | 2 |
| 244 | 1 | 1 | 1 | 1 | 1 | 1 | 1 | 2 | 1 | 1 | 1 | 2 | 2 | 2 | 2 |
| 267 | 1 | 1 | 1 | 1 | 1 | 1 | 2 | 2 | 1 | 1 | 1 | 2 | 2 | 2 | 2 |
| 277 | 1 | 1 | 1 | 1 | 1 | 1 | 2 | 2 | 1 | 1 | 1 | 2 | 2 | 2 | 2 |
| 278 | 1 | 1 | 1 | 1 | 1 | 1 | 1 | 2 | 1 | 1 | 1 | 2 | 1 | 2 | 2 |
| 281 | 1 | 1 | 1 | 1 | 1 | 1 | 1 | 1 | 2 | 1 | 1 | 2 | 2 | 2 | 2 |
| 283 | 1 | 1 | 1 | 1 | 2 | 1 | 1 | 1 | 1 | 1 | 1 | 2 | 2 | 2 | 2 |
| 287 | 1 | 1 | 1 | 1 | 2 | 1 | 1 | 1 | 1 | 1 | 1 | 2 | 2 | 2 | 2 |
| 307 | 1 | 1 | 1 | 1 | 2 | 1 | 2 | 2 | 1 | 1 | 1 | 2 | 2 | 2 | 2 |
| 310 | 1 | 1 | 1 | 1 | 2 | 1 | 1 | 1 | 2 | 1 | 1 | 2 | 2 | 2 | 2 |
| 317 | 1 | 1 | 1 | 2 | 1 | 1 | 1 | 1 | 1 | 1 | 1 | 2 | 2 | 2 | 2 |
| 333 | 1 | 1 | 1 | 1 | 1 | 1 | 2 | 2 | 1 | 1 | 1 | 2 | 2 | 2 | 2 |
| 335 | 1 | 1 | 1 | 1 | 1 | 1 | 2 | 2 | 1 | 1 | 1 | 2 | 2 | 2 | 2 |
| 337 | 1 | 1 | 1 | 1 | 1 | 1 | 2 | 2 | 1 | 1 | 1 | 2 | 2 | 2 | 2 |
| 340 | 1 | 1 | 1 | 1 | 1 | 1 | 2 | 1 | 1 | 1 | 1 | 1 | 2 | 2 | 2 |
| 364 | 1 | 1 | 1 | 1 | 1 | 1 | 1 | 1 | 1 | 1 | 1 | 2 | 2 | 2 | 2 |
| 372 | 1 | 1 | 1 | 1 | 1 | 1 | 1 | 1 | 2 | 1 | 1 | 2 | 2 | 2 | 2 |
| 382 | 1 | 1 | 1 | 1 | 1 | 1 | 2 | 2 | 1 | 1 | 1 | 2 | 2 | 1 | 2 |
| 383 | 1 | 1 | 1 | 2 | 1 | 1 | 2 | 2 | 1 | 1 | 1 | 2 | 2 | 2 | 2 |
| 394 | 1 | 1 | 1 | 1 | 1 | 1 | 2 | 2 | 1 | 1 | 1 | 2 | 2 | 2 | 2 |
| 1 | 1 | 1 | 1 | 1 | 1 | 1 | 1 | 3 | 3 | 1 | 1 | 2 | 3 | 3 | 3 |
| 3 | 1 | 1 | 1 | 1 | 1 | 1 | 2 | 2 | 1 | 2 | 1 | 2 | 3 | 3 | 3 |
| 4 | 1 | 1 | 3 | 3 | 3 | 3 | 3 | 3 | 1 | 1 | 3 | 3 | 3 | 3 | 3 |
| 5 | 1 | 1 | 3 | 3 | 1 | 3 | 3 | 3 | 1 | 1 | 3 | 3 | 3 | 2 | 3 |
| 11 | 1 | 1 | 1 | 1 | 1 | 1 | 1 | 3 | 3 | 1 | 1 | 3 | 3 | 3 | 3 |
| 20 | 1 | 1 | 2 | 3 | 3 | 2 | 3 | 2 | 2 | 1 | 1 | 3 | 3 | 2 | 3 |
| 21 | 1 | 1 | 1 | 1 | 1 | 2 | 3 | 3 | 2 | 1 | 1 | 3 | 3 | 2 | 3 |

Continued table S9.

| **Variety (Line)** | **2020** | | | **2021** | | | | | | **2022** | | **Artificial inoculation** | | | **Resistance level** |
| --- | --- | --- | --- | --- | --- | --- | --- | --- | --- | --- | --- | --- | --- | --- | --- |
|  | **CS-b1** | **CS-b2** | **LC** | **CS-b1** | **CS-b2** | **LC** | **YJ** | **TY** | **SX** | **CS-b1** | **CS-b2** | **No.1** | **No.2** | **No.3** |  |
| 22 | 1 | 1 | 2 | 1 | 1 | 3 | 3 | 3 | 1 | 1 | 1 | 3 | 3 | 2 | 3 |
| 26 | 1 | 1 | 1 | 3 | 3 | 1 | 1 | 3 | 3 | 1 | 1 | 3 | 3 | 2 | 3 |
| 28 | 1 | 1 | 1 | 1 | 1 | 1 | 1 | 3 | 1 | 1 | 1 | 2 | 3 | 3 | 3 |
| 35 | 1 | 1 | 1 | 1 | 1 | 1 | 1 | 3 | 1 | 1 | 1 | 3 | 3 | 2 | 3 |
| 37 | 1 | 1 | 1 | 3 | 1 | 3 | 3 | 3 | 1 | 3 | 1 | 3 | 3 | 2 | 3 |
| 40 | 1 | 1 | 1 | 1 | 1 | 1 | 1 | 2 | 1 | 1 | 1 | 3 | 2 | 3 | 3 |
| 47 | 1 | 1 | 1 | 3 | 1 | 1 | 3 | 2 | 1 | 1 | 3 | 2 | 2 | 3 | 3 |
| 60 | 1 | 1 | 1 | 1 | 1 | 1 | 1 | 3 | 1 | 1 | 1 | 3 | 2 | 3 | 3 |
| 63 | 1 | 1 | 3 | 3 | 1 | 3 | 3 | 3 | 3 | 1 | 1 | 3 | 3 | 3 | 3 |
| 69 | 1 | 1 | 1 | 1 | 1 | 3 | 3 | 2 | 1 | 1 | 1 | 3 | 2 | 3 | 3 |
| 70 | 1 | 1 | 1 | 1 | 1 | 1 | 3 | 3 | 1 | 1 | 1 | 3 | 3 | 3 | 3 |
| 75 | 1 | 1 | 1 | 1 | 1 | 1 | 1 | 3 | 3 | 1 | 1 | 3 | 3 | 3 | 3 |
| 82 | 3 | 1 | 1 | 3 | 3 | 3 | 2 | 3 | 1 | 1 | 3 | 3 | 3 | 3 | 3 |
| 83 | 1 | 1 | 1 | 1 | 1 | 1 | 1 | 3 | 1 | 1 | 1 | 3 | 3 | 3 | 3 |
| 86 | 1 | 1 | 1 | 1 | 1 | 1 | 2 | 3 | 1 | 3 | 1 | 3 | 2 | 3 | 3 |
| 97 | 1 | 1 | 1 | 1 | 1 | 1 | 3 | 3 | 3 | 1 | 1 | 3 | 3 | 3 | 3 |
| 111 | 1 | 1 | 1 | 3 | 3 | 1 | 3 | 3 | 1 | 2 | 1 | 2 | 3 | 3 | 3 |
| 146 | 1 | 1 | 1 | 1 | 1 | 1 | 3 | 3 | 1 | 1 | 1 | 3 | 3 | 2 | 3 |
| 149 | 1 | 1 | 3 | 1 | 2 | 3 | 3 | 2 | 3 | 1 | 1 | 3 | 3 | 3 | 3 |
| 161 | 1 | 1 | 1 | 1 | 1 | 1 | 3 | 3 | 1 | 1 | 1 | 3 | 2 | 3 | 3 |
| 168 | 1 | 1 | 1 | 1 | 1 | 1 | 3 | 3 | 2 | 1 | 1 | 3 | 3 | 2 | 3 |
| 174 | 1 | 1 | 1 | 3 | 1 | 1 | 3 | 3 | 1 | 1 | 1 | 3 | 3 | 3 | 3 |
| 177 | 1 | 1 | 1 | 1 | 1 | 1 | 2 | 3 | 3 | 1 | 1 | 3 | 3 | 3 | 3 |
| 196 | 1 | 1 | 1 | 1 | 1 | 1 | 3 | 3 | 1 | 1 | 1 | 3 | 3 | 3 | 3 |
| 204 | 1 | 1 | 1 | 1 | 1 | 1 | 3 | 3 | 1 | 1 | 1 | 3 | 2 | 3 | 3 |
| 209 | 1 | 1 | 1 | 1 | 1 | 1 | 3 | 3 | 1 | 1 | 1 | 3 | 2 | 3 | 3 |
| 211 | 1 | 1 | 1 | 1 | 1 | 1 | 1 | 3 | 1 | 1 | 1 | 3 | 2 | 3 | 3 |
| 213 | 1 | 1 | 1 | 1 | 1 | 1 | 1 | 3 | 1 | 1 | 1 | 3 | 3 | 3 | 3 |
| 218 | 1 | 1 | 1 | 3 | 3 | 1 | 1 | 3 | 1 | 1 | 1 | 3 | 3 | 3 | 3 |
| 228 | 1 | 1 | 1 | 1 | 1 | 1 | 3 | 1 | 1 | 1 | 1 | 3 | 3 | 3 | 3 |
| 246 | 1 | 1 | 3 | 1 | 1 | 3 | 3 | 3 | 2 | 1 | 1 | 3 | 3 | 3 | 3 |

Continued table S9.

| **Variety (Line)** | **2020** | | | **2021** | | | | | | **2022** | | **Artificial inoculation** | | | **Resistance level** |
| --- | --- | --- | --- | --- | --- | --- | --- | --- | --- | --- | --- | --- | --- | --- | --- |
|  | **CS-b1** | **CS-b2** | **LC** | **CS-b1** | **CS-b2** | **LC** | **YJ** | **TY** | **SX** | **CS-b1** | **CS-b2** | **No.1** | **No.2** | **No.3** |  |
| 248 | 1 | 1 | 1 | 1 | 1 | 3 | 3 | 3 | 1 | 1 | 1 | 3 | 3 | 3 | 3 |
| 254 | 1 | 1 | 1 | 1 | 1 | 1 | 3 | 3 | 1 | 1 | 1 | 3 | 3 | 3 | 3 |
| 274 | 1 | 1 | 1 | 3 | 1 | 1 | 3 | 3 | 1 | 1 | 1 | 3 | 2 | 3 | 3 |
| 276 | 1 | 1 | 1 | 2 | 1 | 3 | 3 | 3 | 1 | 3 | 1 | 3 | 2 | 3 | 3 |
| 282 | 1 | 1 | 1 | 1 | 1 | 1 | 1 | 1 | 3 | 1 | 1 | 3 | 3 | 3 | 3 |
| 293 | 1 | 1 | 1 | 1 | 1 | 1 | 3 | 3 | 1 | 1 | 1 | 3 | 3 | 3 | 3 |
| 294 | 1 | 1 | 1 | 1 | 1 | 1 | 1 | 2 | 3 | 1 | 1 | 2 | 3 | 3 | 3 |
| 298 | 1 | 1 | 1 | 1 | 1 | 1 | 3 | 3 | 1 | 1 | 1 | 3 | 3 | 3 | 3 |
| 301 | 1 | 1 | 1 | 3 | 1 | 1 | 3 | 1 | 3 | 1 | 1 | 3 | 3 | 2 | 3 |
| 305 | 1 | 1 | 1 | 3 | 3 | 1 | 3 | 3 | 2 | 3 | 3 | 3 | 3 | 3 | 3 |
| 306 | 1 | 1 | 1 | 1 | 3 | 1 | 3 | 3 | 1 | 1 | 1 | 3 | 3 | 3 | 3 |
| 309 | 1 | 1 | 1 | 1 | 1 | 1 | 3 | 3 | 1 | 1 | 1 | 3 | 3 | 3 | 3 |
| 319 | 1 | 1 | 1 | 1 | 1 | 1 | 3 | 3 | 1 | 1 | 1 | 2 | 3 | 3 | 3 |
| 320 | 1 | 1 | 1 | 1 | 1 | 1 | 3 | 3 | 1 | 1 | 1 | 3 | 2 | 3 | 3 |
| 328 | 1 | 1 | 1 | 3 | 1 | 1 | 3 | 3 | 1 | 2 | 3 | 3 | 3 | 3 | 3 |
| 343 | 1 | 1 | 1 | 1 | 1 | 1 | 3 | 3 | 1 | 1 | 1 | 3 | 3 | 3 | 3 |
| 345 | 1 | 1 | 1 | 1 | 1 | 1 | 2 | 2 | 2 | 1 | 1 | 3 | 2 | 3 | 3 |
| 348 | 1 | 1 | 1 | 3 | 1 | 1 | 3 | 1 | 1 | 1 | 1 | 3 | 3 | 3 | 3 |
| 351 | 1 | 1 | 1 | 1 | 1 | 1 | 2 | 2 | 1 | 1 | 1 | 3 | 3 | 3 | 3 |
| 357 | 1 | 1 | 1 | 1 | 1 | 1 | 3 | 3 | 1 | 1 | 1 | 3 | 1 | 3 | 3 |
| 367 | 1 | 1 | 1 | 2 | 2 | 1 | 3 | 3 | 2 | 1 | 1 | 2 | 3 | 3 | 3 |
| 368 | 1 | 1 | 1 | 1 | 1 | 1 | 3 | 3 | 3 | 1 | 1 | 3 | 3 | 3 | 3 |
| 370 | 1 | 1 | 3 | 1 | 1 | 1 | 3 | 3 | 1 | 1 | 1 | 3 | 2 | 3 | 3 |
| 375 | 1 | 1 | 1 | 1 | 1 | 1 | 3 | 3 | 1 | 1 | 1 | 3 | 2 | 3 | 3 |
| 387 | 1 | 1 | 1 | 3 | 3 | 1 | 3 | 3 | 1 | 1 | 3 | 3 | 3 | 3 | 3 |
| 389 | 3 | 1 | 1 | 1 | 3 | 1 | 3 | 3 | 1 | 1 | 1 | 3 | 3 | 3 | 3 |
| 390 | 1 | 1 | 1 | 1 | 1 | 1 | 3 | 3 | 3 | 1 | 1 | 3 | 3 | 3 | 3 |
| 395 | 3 | 1 | 1 | 1 | 1 | 1 | 3 | 3 | 1 | 1 | 1 | 3 | 3 | 2 | 3 |
| 396 | 1 | 1 | 1 | 3 | 3 | 1 | 3 | 3 | 1 | 1 | 3 | 2 | 3 | 3 | 3 |
| 398 | 1 | 1 | 1 | 1 | 1 | 1 | 2 | 2 | 1 | 1 | 1 | 3 | 3 | 3 | 3 |
| 399 | 1 | 1 | 1 | 1 | 1 | 1 | 3 | 3 | 3 | 1 | 1 | 3 | 3 | 3 | 3 |

Continued table S9.

| **Variety (Line)** | **2020** | | | **2021** | | | | | | **2022** | | **Artificial inoculation** | | | **Resistance level** |
| --- | --- | --- | --- | --- | --- | --- | --- | --- | --- | --- | --- | --- | --- | --- | --- |
|  | **CS-b1** | **CS-b2** | **LC** | **CS-b1** | **CS-b2** | **LC** | **YJ** | **TY** | **SX** | **CS-b1** | **CS-b2** | **No.1** | **No.2** | **No.3** |  |
| 400 | 1 | 1 | 1 | 1 | 1 | 1 | 3 | 3 | 1 | 3 | 1 | 3 | 3 | 3 | 3 |
| 29 | 1 | 1 | 4 | 1 | 1 | 1 | 1 | 4 | 1 | 1 | 1 | 3 | 4 | 4 | 4 |
| 38 | 1 | 1 | 4 | 1 | 1 | 3 | 1 | 4 | 1 | 1 | 1 | 4 | 4 | 4 | 4 |
| 44 | 1 | 1 | 3 | 4 | 1 | 3 | 4 | 4 | 3 | 1 | 1 | 4 | 3 | 4 | 4 |
| 49 | 1 | 1 | 3 | 3 | 1 | 4 | 4 | 4 | 1 | 1 | 1 | 4 | 4 | 4 | 4 |
| 59 | 1 | 1 | 4 | 1 | 1 | 4 | 4 | 3 | 1 | 1 | 1 | 4 | 4 | 4 | 4 |
| 68 | 1 | 1 | 4 | 4 | 1 | 4 | 4 | 4 | 3 | 1 | 1 | 3 | 4 | 4 | 4 |
| 71 | 1 | 1 | 1 | 1 | 1 | 1 | 3 | 4 | 1 | 1 | 1 | 3 | 4 | 4 | 4 |
| 72 | 1 | 1 | 1 | 1 | 1 | 1 | 3 | 4 | 2 | 1 | 1 | 4 | 3 | 4 | 4 |
| 76 | 1 | 1 | 1 | 1 | 1 | 1 | 4 | 4 | 3 | 1 | 1 | 4 | 3 | 4 | 4 |
| 79 | 1 | 1 | 1 | 1 | 1 | 1 | 2 | 4 | 1 | 1 | 1 | 4 | 3 | 4 | 4 |
| 80 | 1 | 1 | 4 | 1 | 1 | 4 | 4 | 4 | 3 | 1 | 1 | 3 | 4 | 4 | 4 |
| 81 | 1 | 1 | 4 | 1 | 1 | 4 | 4 | 4 | 3 | 1 | 1 | 4 | 3 | 4 | 4 |
| 84 | 1 | 1 | 1 | 4 | 1 | 4 | 4 | 2 | 1 | 1 | 4 | 4 | 3 | 4 | 4 |
| 85 | 3 | 3 | 3 | 3 | 4 | 3 | 3 | 3 | 3 | 3 | 3 | 3 | 4 | 4 | 4 |
| 91 | 1 | 1 | 1 | 1 | 1 | 3 | 1 | 3 | 3 | 4 | 1 | 3 | 4 | 4 | 4 |
| 92 | 1 | 1 | 1 | 1 | 1 | 4 | 3 | 4 | 3 | 1 | 1 | 4 | 2 | 3 | 4 |
| 99 | 1 | 1 | 3 | 1 | 1 | 3 | 4 | 4 | 4 | 1 | 1 | 3 | 4 | 4 | 4 |
| 113 | 4 | 1 | 1 | 4 | 1 | 1 | 3 | 4 | 1 | 3 | 3 | 3 | 4 | 4 | 4 |
| 119 | 1 | 1 | 1 | 4 | 1 | 1 | 3 | 3 | 3 | 1 | 1 | 4 | 4 | 4 | 4 |
| 145 | 1 | 1 | 1 | 1 | 1 | 1 | 4 | 4 | 3 | 1 | 1 | 4 | 4 | 4 | 4 |
| 162 | 1 | 1 | 4 | 4 | 1 | 4 | 4 | 4 | 1 | 2 | 1 | 3 | 4 | 4 | 4 |
| 164 | 1 | 1 | 1 | 1 | 1 | 1 | 4 | 4 | 4 | 1 | 1 | 3 | 4 | 4 | 4 |
| 165 | 1 | 1 | 1 | 1 | 1 | 4 | 3 | 1 | 1 | 1 | 1 | 4 | 4 | 4 | 4 |
| 170 | 3 | 1 | 1 | 3 | 1 | 1 | 4 | 4 | 2 | 1 | 1 | 4 | 4 | 4 | 4 |
| 172 | 1 | 1 | 1 | 1 | 1 | 1 | 4 | 4 | 1 | 1 | 1 | 4 | 3 | 4 | 4 |
| 178 | 1 | 1 | 4 | 1 | 1 | 4 | 3 | 3 | 1 | 1 | 1 | 4 | 4 | 4 | 4 |
| 180 | 1 | 1 | 4 | 4 | 2 | 4 | 3 | 3 | 1 | 1 | 2 | 4 | 3 | 4 | 4 |
| 184 | 1 | 1 | 3 | 1 | 1 | 4 | 4 | 4 | 3 | 1 | 1 | 4 | 4 | 4 | 4 |
| 199 | 1 | 1 | 1 | 1 | 1 | 1 | 4 | 4 | 1 | 1 | 1 | 4 | 3 | 4 | 4 |
| 200 | 3 | 3 | 4 | 3 | 3 | 4 | 4 | 4 | 4 | 3 | 3 | 3 | 4 | 4 | 4 |

Continued table S9.

| **Variety (Line)** | **2020** | | | **2021** | | | | | | **2022** | | **Artificial inoculation** | | | **Resistance level** |
| --- | --- | --- | --- | --- | --- | --- | --- | --- | --- | --- | --- | --- | --- | --- | --- |
|  | **CS-b1** | **CS-b2** | **LC** | **CS-b1** | **CS-b2** | **LC** | **YJ** | **TY** | **SX** | **CS-b1** | **CS-b2** | **No.1** | **No.2** | **No.3** |  |
| 201 | 1 | 1 | 1 | 1 | 1 | 1 | 4 | 4 | 3 | 1 | 1 | 4 | 3 | 4 | 4 |
| 212 | 1 | 1 | 1 | 3 | 1 | 1 | 3 | 4 | 1 | 1 | 3 | 4 | 4 | 4 | 4 |
| 223 | 1 | 1 | 4 | 1 | 1 | 4 | 4 | 4 | 4 | 1 | 1 | 4 | 4 | 4 | 4 |
| 229 | 1 | 1 | 1 | 4 | 4 | 1 | 4 | 4 | 1 | 1 | 1 | 4 | 4 | 4 | 4 |
| 239 | 1 | 1 | 1 | 4 | 1 | 1 | 3 | 4 | 1 | 4 | 1 | 4 | 4 | 4 | 4 |
| 240 | 1 | 1 | 1 | 1 | 1 | 1 | 4 | 4 | 1 | 1 | 1 | 4 | 3 | 4 | 4 |
| 256 | 1 | 1 | 1 | 3 | 3 | 1 | 3 | 4 | 1 | 1 | 1 | 4 | 4 | 4 | 4 |
| 258 | 1 | 1 | 4 | 3 | 1 | 4 | 4 | 2 | 2 | 1 | 1 | 4 | 4 | 4 | 4 |
| 261 | 1 | 1 | 1 | 1 | 1 | 1 | 4 | 4 | 1 | 2 | 1 | 4 | 3 | 3 | 4 |
| 270 | 1 | 1 | 1 | 1 | 1 | 4 | 3 | 3 | 1 | 1 | 1 | 4 | 3 | 4 | 4 |
| 273 | 1 | 1 | 1 | 1 | 1 | 1 | 4 | 4 | 4 | 1 | 1 | 4 | 4 | 4 | 4 |
| 275 | 1 | 1 | 1 | 1 | 1 | 1 | 3 | 4 | 1 | 1 | 1 | 4 | 3 | 4 | 4 |
| 288 | 3 | 1 | 1 | 1 | 1 | 1 | 3 | 4 | 2 | 1 | 1 | 4 | 4 | 4 | 4 |
| 289 | 3 | 1 | 4 | 1 | 1 | 1 | 2 | 2 | 1 | 1 | 1 | 4 | 3 | 4 | 4 |
| 292 | 1 | 1 | 1 | 4 | 4 | 1 | 4 | 4 | 3 | 1 | 1 | 4 | 4 | 4 | 4 |
| 297 | 1 | 1 | 4 | 1 | 1 | 4 | 2 | 2 | 1 | 3 | 1 | 3 | 4 | 4 | 4 |
| 302 | 1 | 1 | 4 | 1 | 1 | 1 | 4 | 4 | 1 | 1 | 1 | 4 | 2 | 4 | 4 |
| 308 | 1 | 1 | 1 | 1 | 1 | 4 | 3 | 3 | 1 | 1 | 1 | 4 | 3 | 4 | 4 |
| 315 | 1 | 1 | 1 | 3 | 4 | 1 | 2 | 2 | 1 | 1 | 3 | 4 | 3 | 4 | 4 |
| 318 | 1 | 1 | 1 | 1 | 4 | 1 | 2 | 2 | 1 | 1 | 1 | 4 | 2 | 4 | 4 |
| 322 | 1 | 1 | 1 | 3 | 4 | 1 | 3 | 4 | 2 | 1 | 3 | 4 | 3 | 4 | 4 |
| 323 | 1 | 1 | 4 | 1 | 1 | 1 | 4 | 4 | 1 | 1 | 1 | 4 | 3 | 4 | 4 |
| 334 | 1 | 1 | 1 | 1 | 1 | 4 | 3 | 2 | 1 | 1 | 1 | 3 | 4 | 4 | 4 |
| 339 | 4 | 4 | 1 | 4 | 1 | 1 | 3 | 1 | 4 | 3 | 1 | 4 | 4 | 4 | 4 |
| 359 | 1 | 1 | 1 | 1 | 1 | 1 | 4 | 4 | 1 | 1 | 1 | 4 | 4 | 4 | 4 |
| 361 | 3 | 3 | 3 | 4 | 4 | 3 | 4 | 4 | 3 | 3 | 4 | 4 | 3 | 4 | 4 |
| 363 | 1 | 1 | 1 | 1 | 1 | 1 | 4 | 4 | 1 | 1 | 1 | 1 | 4 | 4 | 4 |
| 366 | 1 | 1 | 1 | 1 | 3 | 1 | 4 | 4 | 1 | 1 | 1 | 4 | 2 | 4 | 4 |
| 380 | 1 | 1 | 4 | 1 | 1 | 1 | 4 | 4 | 1 | 1 | 1 | 4 | 3 | 4 | 4 |
| 386 | 1 | 1 | 1 | 1 | 1 | 1 | 4 | 4 | 3 | 1 | 1 | 4 | 3 | 4 | 4 |
| 391 | 1 | 1 | 1 | 1 | 1 | 1 | 3 | 4 | 4 | 1 | 1 | 4 | 2 | 4 | 4 |

Continued table S9.

| **Variety (Line)** | **2020** | | | **2021** | | | | | | **2022** | | **Artificial inoculation** | | | **Resistance level** |
| --- | --- | --- | --- | --- | --- | --- | --- | --- | --- | --- | --- | --- | --- | --- | --- |
|  | **CS-b1** | **CS-b2** | **LC** | **CS-b1** | **CS-b2** | **LC** | **YJ** | **TY** | **SX** | **CS-b1** | **CS-b2** | **No.1** | **No.2** | **No.3** |  |
| 403 | 1 | 1 | 1 | 1 | 1 | 1 | 3 | 3 | 3 | 3 | 1 | 3 | 4 | 4 | 4 |
| 23 | 4 | 3 | 5 | 5 | 4 | 5 | 5 | 5 | 4 | 1 | 5 | 5 | 5 | 4 | 5 |
| 27 | 1 | 1 | 5 | 5 | 1 | 5 | 5 | 5 | 4 | 1 | 5 | 5 | 4 | 5 | 5 |
| 32 | 4 | 4 | 3 | 1 | 1 | 4 | 4 | 4 | 4 | 1 | 1 | 4 | 5 | 4 | 5 |
| 61 | 4 | 3 | 1 | 5 | 1 | 1 | 4 | 4 | 3 | 4 | 4 | 5 | 4 | 4 | 5 |
| 62 | 5 | 5 | 5 | 5 | 5 | 5 | 5 | 5 | 4 | 5 | 4 | 5 | 4 | 5 | 5 |
| 88 | 1 | 1 | 4 | 5 | 1 | 5 | 1 | 4 | 1 | 4 | 5 | 5 | 5 | 4 | 5 |
| 98 | 1 | 1 | 5 | 5 | 1 | 4 | 5 | 3 | 4 | 4 | 1 | 4 | 5 | 5 | 5 |
| 103 | 1 | 1 | 5 | 1 | 1 | 4 | 4 | 5 | 4 | 1 | 1 | 4 | 4 | 5 | 5 |
| 105 | 1 | 1 | 1 | 5 | 1 | 1 | 4 | 5 | 2 | 1 | 2 | 5 | 4 | 4 | 5 |
| 108 | 1 | 1 | 1 | 1 | 1 | 1 | 4 | 5 | 2 | 1 | 1 | 5 | 5 | 3 | 5 |
| 110 | 1 | 1 | 3 | 1 | 1 | 3 | 4 | 5 | 4 | 1 | 1 | 5 | 4 | 4 | 5 |
| 124 | 1 | 1 | 1 | 5 | 5 | 1 | 5 | 4 | 1 | 1 | 5 | 5 | 5 | 4 | 5 |
| 130 | 4 | 1 | 4 | 5 | 5 | 5 | 5 | 5 | 4 | 5 | 5 | 5 | 4 | 5 | 5 |
| 134 | 4 | 4 | 5 | 5 | 5 | 5 | 5 | 5 | 3 | 5 | 3 | 5 | 4 | 5 | 5 |
| 138 | 5 | 5 | 4 | 1 | 1 | 5 | 5 | 5 | 4 | 1 | 1 | 5 | 5 | 5 | 5 |
| 142 | 5 | 5 | 1 | 5 | 5 | 4 | 5 | 4 | 4 | 5 | 5 | 5 | 4 | 5 | 5 |
| 159 | 1 | 1 | 5 | 1 | 1 | 5 | 4 | 5 | 3 | 1 | 1 | 5 | 5 | 5 | 5 |
| 171 | 3 | 1 | 1 | 5 | 1 | 1 | 5 | 1 | 1 | 5 | 5 | 5 | 4 | 5 | 5 |
| 173 | 1 | 1 | 5 | 1 | 1 | 4 | 4 | 3 | 1 | 1 | 1 | 5 | 5 | 5 | 5 |
| 179 | 1 | 1 | 4 | 5 | 1 | 5 | 4 | 4 | 2 | 4 | 1 | 5 | 4 | 5 | 5 |
| 191 | 5 | 1 | 1 | 5 | 1 | 1 | 4 | 5 | 1 | 5 | 2 | 5 | 5 | 4 | 5 |
| 192 | 4 | 2 | 5 | 5 | 5 | 5 | 4 | 5 | 1 | 5 | 3 | 5 | 4 | 4 | 5 |
| 210 | 1 | 1 | 1 | 1 | 1 | 1 | 5 | 5 | 1 | 1 | 1 | 5 | 5 | 5 | 5 |
| 230 | 1 | 1 | 1 | 1 | 1 | 1 | 5 | 5 | 1 | 1 | 1 | 5 | 4 | 5 | 5 |
| 231 | 5 | 5 | 1 | 4 | 4 | 1 | 4 | 5 | 1 | 1 | 4 | 5 | 5 | 5 | 5 |
| 236 | 5 | 1 | 1 | 5 | 1 | 1 | 4 | 5 | 1 | 5 | 5 | 5 | 4 | 5 | 5 |
| 247 | 1 | 1 | 3 | 4 | 1 | 5 | 4 | 4 | 2 | 4 | 1 | 5 | 5 | 5 | 5 |
| 252 | 1 | 1 | 1 | 3 | 4 | 1 | 5 | 5 | 3 | 1 | 3 | 5 | 4 | 4 | 5 |
| 255 | 5 | 5 | 5 | 5 | 5 | 5 | 5 | 5 | 5 | 5 | 5 | 5 | 4 | 5 | 5 |
| 268 | 5 | 5 | 5 | 5 | 5 | 5 | 5 | 3 | 5 | 5 | 5 | 5 | 4 | 5 | 5 |

Continued table S9.

| **Variety (Line)** | **2020** | | | **2021** | | | | | | **2022** | | **Artificial inoculation** | | | **Resistance level** |
| --- | --- | --- | --- | --- | --- | --- | --- | --- | --- | --- | --- | --- | --- | --- | --- |
|  | **CS-b1** | **CS-b2** | **LC** | **CS-b1** | **CS-b2** | **LC** | **YJ** | **TY** | **SX** | **CS-b1** | **CS-b2** | **No.1** | **No.2** | **No.3** |  |
| 269 | 4 | 5 | 5 | 3 | 5 | 5 | 3 | 4 | 3 | 5 | 3 | 5 | 5 | 3 | 5 |
| 295 | 1 | 1 | 1 | 1 | 1 | 1 | 4 | 5 | 4 | 1 | 1 | 5 | 5 | 5 | 5 |
| 300 | 4 | 4 | 5 | 5 | 4 | 5 | 5 | 5 | 4 | 5 | 5 | 5 | 3 | 5 | 5 |
| 324 | 3 | 1 | 3 | 5 | 1 | 4 | 5 | 4 | 2 | 5 | 5 | 4 | 4 | 5 | 5 |
| 330 | 4 | 4 | 3 | 5 | 4 | 3 | 5 | 4 | 3 | 3 | 2 | 4 | 5 | 5 | 5 |
| 341 | 5 | 5 | 5 | 5 | 5 | 5 | 5 | 5 | 5 | 5 | 5 | 5 | 3 | 5 | 5 |
| 342 | 5 | 5 | 5 | 5 | 5 | 4 | 5 | 5 | 4 | 5 | 5 | 4 | 5 | 5 | 5 |
| 384 | 5 | 4 | 1 | 5 | 5 | 1 | 5 | 5 | 1 | 4 | 3 | 5 | 4 | 5 | 5 |
| 385 | 3 | 1 | 1 | 4 | 1 | 1 | 4 | 3 | 1 | 2 | 4 | 4 | 5 | 5 | 5 |
| 393 | 4 | 4 | 5 | 4 | 5 | 5 | 4 | 5 | 3 | 5 | 4 | 5 | 4 | 5 | 5 |

**Table S10.** Correlation coefficients of SMD response data of the F_1_ progeny population under different environments

| **Environment** | **CS-b1**  **2020** | **CS-b2**  **2020** | **CS-b1**  **2021** | **CS-b2**  **2021** | **CS-b1**  **2022** | **CS-b2**  **2022** | **LC 2020** | **LC 2021** | **YJ 2021** | **TY 2021** | **SX 2021** | **AIT-1** | **AIT-2** | **AIT-3** |
| --- | --- | --- | --- | --- | --- | --- | --- | --- | --- | --- | --- | --- | --- | --- |
| CS-b1 2020 | 1.00*** |  |  |  |  |  |  |  |  |  |  |  |  |  |
| CS-b2 2020 | 0.84*** | 1.00*** |  |  |  |  |  |  |  |  |  |  |  |  |
| CS-b1 2021 | 0.58*** | 0.47*** | 1.00*** |  |  |  |  |  |  |  |  |  |  |  |
| CS-b2 2021 | 0.52*** | 0.57*** | 0.53*** | 1.00*** |  |  |  |  |  |  |  |  |  |  |
| CS-b1 2022 | 0.70*** | 0.59*** | 0.65*** | 0.45*** | 1.00*** |  |  |  |  |  |  |  |  |  |
| CS-b2 2022 | 0.64*** | 0.53*** | 0.74*** | 0.56*** | 0.63*** | 1.00*** |  |  |  |  |  |  |  |  |
| LC 2020 | 0.36*** | 0.42*** | 0.38*** | 0.28*** | 0.41*** | 0.38*** | 1.00*** |  |  |  |  |  |  |  |
| LC 2021 | 0.39*** | 0.45*** | 0.44*** | 0.29*** | 0.48*** | 0.44*** | 0.78*** | 1.00*** |  |  |  |  |  |  |
| YJ 2021 | 0.44*** | 0.39*** | 0.50*** | 0.29*** | 0.41*** | 0.46*** | 0.48*** | 0.49*** | 1.00*** |  |  |  |  |  |
| TY 2021 | 0.38*** | 0.32*** | 0.39*** | 0.28*** | 0.35*** | 0.38*** | 0.44*** | 0.40*** | 0.79*** | 1.00*** |  |  |  |  |
| SX 2021 | 0.41*** | 0.50*** | 0.34*** | 0.30*** | 0.35*** | 0.33*** | 0.45*** | 0.47*** | 0.47*** | 0.46*** | 1.00*** |  |  |  |
| AIT-1 | 0.46*** | 0.38*** | 0.54*** | 0.34*** | 0.45*** | 0.48*** | 0.47*** | 0.50*** | 0.79*** | 0.82*** | 0.47*** | 1.00*** |  |  |
| AIT-2 | 0.37*** | 0.30*** | 0.45*** | 0.26*** | 0.35*** | 0.36*** | 0.41*** | 0.45*** | 0.69*** | 0.71*** | 0.45*** | 0.79*** | 1.00*** |  |
| AIT-3 | 0.45*** | 0.38*** | 0.51*** | 0.31*** | 0.45*** | 0.46*** | 0.51*** | 0.51*** | 0.81*** | 0.81*** | 0.49*** | 0.91*** | 0.81*** | 1.00*** |

*Note:*** Significant correlation at p < 0.001. CS: Cangshan, LC: Longchuan, SX: Suixi, TY: Tianyang, YJ: Yuanjiang, AIT: artificial inoculation treatment, b: block.*

**Table S11.** Nucleotide sequence of probes targeting the QTLs related to SMD resistance

| **QTL name** | **SNP Marker ID** | **Probe sequence with** SNP variation site shown in red letters |
| --- | --- | --- |
| *qRsm-Y12* | AX-171367442 | CAAGGCAACATGTAAATAATGATGGCAGACATTGT[T/C]TGGCTCAGAATGAGGCTCAGCAAAATATTGCTCCC |
|  | AX-171312668 | TCTCTTCGTAACGCATAGACCTCCTAGCAAATGAC[A/G]AAAATATAAGCCATACACTACACATCAAACAAACA |
| *qRsm-Y41* | AX-171308038 | CAACTTCAGATACAAGCAAGAAAACAGAACTTCTC[A/G]CATTCAAATGTACTGTACACTTAAGGGTTCAATAT |
|  | AX-171265900 | TTATTTTTATGTAGCGTGATTCATTTTTGCAATCA[A/G]CTCATTTCCTGTAATTCCTCATCTAATGCTGCTGA |
| *qRsm-Y52* | AX-171266761 | AGTGAGTTCAAATATTTGATCTCCTTTTGGACATA[T/G]GGCACCTCAGATTTGAATACTTTTCTAATTGATTA |
|  | AX-117172243 | TTAATACACTTGGTCCTAATCAAGTAATTATAACC[A/G]TCTGAACTTGTTTCAACAGGAGTGCTGCATTATGG |
| *qRsm-Y75* | AX-171332119 | ACATGAATAACAGATCTGGTACAGGCTCTCCTTGT[T/C]TAGCCCTGACGATTCCTTTTGATAGACTGAACTTT |
|  | AX-171288089 | TTCTTGAGACGAGTTGTTCCCCCCTCTTTGCTTAT[T/C]GGTATTTTCATGTGTCCAAAAGGAGTGTTGACATC |
| *qRsm-R14* | AX-171290689 | TAGCATGGAAAACTGGAGCATTATTCAAATCAGGA[A/G]TGGCATGGCCTTCCAAGCCAGTTGAATGCCGATGA |
|  | AX-171329853 | TTGAAATCAATGGGAAAGTTTTCCAAGGTGCTACA[A/G]CTTTTGATTTTCTATTGTGCCAAACAATATAATGT |
| *qRsm-R23* | AX-171330585 | GTCAATATAAAATTTCGTCAGTTGCTGTTTACATG[T/G]ATTGAATTCATGCCCATGTCCATTTGACTCTTCTG |
|  | AX-171286409 | ATGCGAGTTAGTTTCACCATGTAAATCAGCCATAA[A/G]ATGATTCCCAAGGTAGTGATAAACTTTGCAAGTAG |
| *qRsm-R92* | AX-171360287 | TTTCATATCGTTTGATTGGGCTATTTTTGCACCAC[T/C]TGGCGAACCTTGCATTGCATGATAGACTACACTAC |
|  | AX-171296656 | AAAGGTTCAAATGCTGAAATCTCCATCGTTCCAGT[T/C]ATCTAATAAGCGGTCTGCACAGAAGGAGCCACCGT |

**Table S12.** Candidate genes related to disease resistance based on plant pathogen receptor genes database and plant transcription factor database

| **QTL Name** | **HG** | **Left Marker Position** | **Right Marker Position** | **PRG** | **TFG** |
| --- | --- | --- | --- | --- | --- |
| *qRsm-Y12* | HG05 | Chr05D7687932 | Chr05D10075423 | 8 | 2 |
| *qRsm-Y41* | HG09 | Chr09D48846978 | Chr09D55497325 | 8 | 6 |
| *qRsm-Y52* | HG09 | Chr09C46532044 | Chr09C71247725 | 54 | 35 |
| *qRsm-Y57* | HG02 | Chr02A26232132 | Chr02A27659566 | 0 | 0 |
| *qRsm-R14* | HG07 | Chr07C49437891 | Chr07C51097686 | 14 | 6 |
| *qRsm-R23* | HG03 | Chr03C22237776 | Chr03C39955456 | 22 | 18 |
| *qRsm-R92* | HG04 | Chr04F30825683 | Chr04F33140683 | 4 | 2 |

*Note: HG: Homo(eo)logous group; PRG: pathogen receptor genes; TFG: transcription factor genes.*
